# Arbuscular mycorrhizal fungi-mediated activation of plant defense responses in direct seeded rice (*Oryza sativa* L.) against root-knot nematode *Meloidogyne graminicola*

**DOI:** 10.3389/fmicb.2023.1104490

**Published:** 2023-05-02

**Authors:** Deepti Malviya, Prakash Singh, Udai B. Singh, Surinder Paul, Pradeep Kumar Bisen, Jai P. Rai, Ram Lakhan Verma, R. Abdul Fiyaz, A. Kumar, Poonam Kumari, Sailabala Dei, Mohd. Reyaz Ahmed, D. J. Bagyaraj, Harsh V. Singh

**Affiliations:** ^1^Plant-Microbe Interaction and Rhizosphere Biology Lab, ICAR-National Bureau of Agriculturally Important Microorganisms, Maunath Bhanjan, India; ^2^Department of Plant Breeding and Genetics, Veer Kunwar Singh College of Agriculture, Bihar Agricultural University, Dumraon, India; ^3^Krishi Vigyan Kendra, Gola Gokaran Nath, India; ^4^Department of Mycology and Plant Pathology, Institute of Agricultural Sciences, Banaras Hindu University, Varanasi, India; ^5^Division of Crop Improvement, ICAR-National Rice Research Institute, Cuttack, India; ^6^Division of Crop Improvement, ICAR-Indian Institute of Rice Research, Hyderabad, India; ^7^Bihar Agricultural University, Bhagalpur, India; ^8^Agrotechnology Division, CSIR-Institute of Himalayan Bioresource Technology, Palampur, India; ^9^Department of Plant Pathology, Veer Kunwar Singh College of Agriculture, Bihar Agricultural University, Dumraon, India; ^10^Centre for Natural Biological Resources and Community Development, Bengaluru, India

**Keywords:** AM fungi, root-knot nematode, rice (*Oryza sativa* L.), *Meloidogyne graminicola*, *Funneliformis mosseae*, *Rhizophagus fasciculatus*, *Rhizophagus intraradices*, plant defense

## Abstract

Rhizosphere is the battlefield of beneficial and harmful (so called phytopathogens) microorganisms. Moreover, these microbial communities are struggling for their existence in the soil and playing key roles in plant growth, mineralization, nutrient cycling and ecosystem functioning. In the last few decades, some consistent pattern have been detected so far that link soil community composition and functions with plant growth and development; however, it has not been studied in detail. AM fungi are model organisms, besides potential role in nutrient cycling; they modulate biochemical pathways directly or indirectly which lead to better plant growth under biotic and abiotic stress conditions. In the present investigations, we have elucidated the AM fungi-mediated activation of plant defense responses against *Meloidogyne graminicola* causing root-knot disease in direct seeded rice (*Oryza sativa* L.). The study describes the multifarious effects of *Funneliformis mosseae, Rhizophagus fasciculatus*, and *Rhizophagus intraradices* inoculated individually or in combination under glasshouse conditions in rice plants. It was found that *F. mosseae, R. fasciculatus* and *R. intraradices* when applied individually or in combination modulated the biochemical and molecular mechanisms in the susceptible and resistant inbred lines of rice. AM inoculation significantly increased various plant growth attributes in plants with simultaneous decrease in the root-knot intensity. Among these, the combined application of *F. mosseae, R. fasciculatus*, and *R. intraradices* was found to enhance the accumulation and activities of biomolecules and enzymes related to defense priming as well as antioxidation in the susceptible and resistant inbred lines of rice pre-challenged with *M. graminicola*. The application of *F. mosseae, R. fasciculatus* and *R. intraradices*, induced the key genes involved in plant defense and signaling and it has been demonstrated for the first time. Results of the present investigation advocated that the application of *F. mosseae, R. fasciculatus* and *R. intraradices*, particularly a combination of all three, not only helped in the control of root-knot nematodes but also increased plant growth as well as enhances the gene expression in rice. Thus, it proved to be an excellent biocontrol as well as plant growth-promoting agent in rice even when the crop is under biotic stress of the root-knot nematode, *M. graminicola*.

## Introduction

Rice (*Oryza sativa* L.) is one of the most important and staple food crops in South East Asia including India plays a vital role in the food security, livelihood wellness and country’s economy (Mahajan et al., 2017). Rice is grown on an area of 43.78 mha in India with an annual production of 127.9 million tonnes approximately (Anoymous, 2022). Direct-seeded rice covers 23% of the area worldwide (Rao et al., 2007; Marasini et al., 2016; Devaraja et al., 2017). Aside from the benefits of direct-seeded rice, the productivity of direct-seeded rice is hindered by various biotic and abiotic stresses (Singh et al., 2012a,b,c, 2016, 2021). Nematode infestation is one of the major biotic factors that can cause yield losses of up to 72% in rice (Khan and Ahamad, 2020). *Ditylenchus angustus*, *Meloidogyne graminicola*, *Aphelenchoides besseyi*, *Hirschmanniella* spp. and *Heterodera oryzicola* are the prevalent nematode species which invade rice (Walia and Bajaj, 2003; Wesemael et al., 2011; Singh et al., 2012c). Among them, *M. graminicola*, an endo-parasitic sedentary nematode causing root-knot disease is the most notorious pathogen of rice. It has been reported from every rice-producing regions of the world and causes a 10.54 per cent production loss in direct-seeded rice in India, costing about 779.30 million rupees every year (Singh et al., 2007; Kumari et al., 2016; Zhou et al., 2020). When environmental conditions are favorable, the infectious juvenile stage (J_2_) emerges from the egg, locates the root, and moves into the meristematic zone and feeds continuously to stimulate the production of huge galls which leads to impairment in the nutrient and water uptake and translocation (Devaraja et al., 2017; Sacchi et al., 2021). *M. graminicola* is an obligate biotroph causing stunting, wilting and decreased tillering; juvenile plants show chlorosis, and mature spikelets with empty florets, resulting in a substantial loss in growth and yield in rice leading to huge losses of foreign exchange (Singh et al., 2007; Jain et al., 2012; Khan and Ahamad, 2020). The direct impairment caused by root-knot nematodes can be intensified by secondary infections of the wounded plant tissues by other pathogens (Walia and Bajaj, 2003; De Waele and Elsen, 2007; Upadhyay and Dwivedi, 2008).

Conventionally root-knot nematode (RKN) is controlled by integrating chemical nematicides with cultural methods and resistant cultivars (Walia and Bajaj, 2003). Chemical nematicides are very expensive and have a negative impact on the useful microorganisms and fauna found in agricultural soil as well as could lead to the development of resistant pathogenic strains (Walia and Bajaj, 2003; Singh et al., 2007, 2012a; Upadhyay and Dwivedi, 2008). In recent decades, a number of compounds, including methyl bromide and aldicarb, have been taken off the market because of risks to the environment, human health, and non-target organisms (Kim et al., 2018; Xiang et al., 2018). Resistance development in the pathogens, residual toxicity of chemical nematicides on the environment and animal health, with the possible withdrawal of pesticides specially nematicides and soil fumigants from the schedule of pesticides and the demand for residue-free produce have obligated researchers and rice growers to explore suitable alternatives strategies for the management of RKN in agriculturally important crops including rice (Singh et al., 2007, 2012a,2012b,2012c, 2013a,2013b, 2017). Among the possible alternatives, the development of resistant cultivars with a high degree of resistance to RKN is of great importance (Walia and Bajaj, 2003; Kumari et al., 2016; Zhou et al., 2020). Moreover, availability of resistance genes in the suitable donor parents is not an easy task. As the resistance is polygenic which further increases the difficulties associated with resistance breeding programme. Further, the pyramiding/transfer of desired gene(s)/quantitative trait loci (QTLs) into commercial cultivars using a resistance breeding programme is a great challenge to rice breeders (Singh et al., 2013a; Forghani and Hajihassani, 2020; Dash et al., 2021; Sun et al., 2021). Under these circumstances, the use of microbe-based strategies is a more environment-friendly, residue-free, safer, and emerging approach to combat RKN effectively (Walia and Bajaj, 2003; Singh et al., 2012a,b,c, 2013a,2013b, 2017). In recent past, several workers evaluated and used biological control agents of microbial origin to control RKN in many crops including rice. Among them, fungal bioagents (*Arthrobotrys oligospora, Monacrosporium eudermatum, Drechslerella dactyloides, Dactylaria brochopaga, Trichoderma harzianum, T. asperellum, T. virens*), and bacterial (*Bacillus subtilis, Paecilomyces lilacinus, B. licheniformis, B. cereus, Pseudomonas fluorescens*, and *Streptomyces cacaoi*) are noteworthy (Singh et al., 2012a,b,c, 2013a,2013b, 2017, 2020; Abd-Elgawad and Askary, 2018; Haque et al., 2018; Zhao et al., 2018; DiLegge et al., 2019; Molinari and Leonetti, 2019; Seo et al., 2019; Singh U. B. et al., 2019; Forghani and Hajihassani, 2020; Sun et al., 2021). Similarly, few studied were made on the application of AM fungi for controlling nematodes in agriculturally important crops (van der Heijden et al., 2015; Bagyaraj et al., 2022).

Recently, several studies reported that arbuscular mycorrhizal fungi (AMF) may offer a safer alternative to pesticide use as the demand for environmental and agricultural safety grows (Harrier and Watson, 2004; Dong et al., 2006; Yang et al., 2014; Bagyaraj et al., 2022). AMF, which are found naturally in soil and behave as bio-stimulators and bio-protectors, may be extremely advantageous to sustainable agriculture by preserving plant productivity and alleviating soil-borne plant pathogens while causing no harm to the environment (Newsham et al., 1995; Smith and Smith, 2011; Koffi et al., 2013; da Silva Campos, 2020). Though there are different kinds of mycorrhiza, the most common mycorrhizal association occurring in crops important in agriculture is the arbuscular type (Babikova et al., 2013; Ceballos et al., 2013; van der Heijden et al., 2015; Bagyaraj et al., 2022). AMF are obligate root symbionts, appraised to colonize more than 80% of all land plant species and they are beneficial for the growth of host plants (van der Heijden et al., 2015). In exchange for photosynthetic carbon from their host, they boost plant growth and development by enhancing nutrient uptake (Bonfante and Genre, 2010; Smith et al., 2010; Bender et al., 2015; Bagyaraj et al., 2022) and also help plants to cope with various stresses imposed by abiotic and biotic elements, including parasitic nematodes on plants (Singh et al., 2012c; Schouteden et al., 2015; van der Heijden et al., 2015). Typically found in the rhizosphere, phytopathogenic nematodes including RKN colonize the roots of their host plants and have opposing effects on the health of those plants (Kumari et al., 2016; Zhou et al., 2020). However, few reports indicated that AMF inoculation reduces the infestation of plant roots by phytopathogenic nematodes (Agrios, 2005; Elsen et al., 2008). Growing plants with AMF inoculation in the nursery can increase their growth and safeguard them from infection caused by soil-borne phytopathogens including phytopathogenic nematodes (Elsen et al., 2008). The AMF can function as biocontrol agents through direct or indirect processes, increasing nutrient uptake and mitigating the harm caused by nematodes (Bagyaraj and Gautam Chawla, 2012). Direct impacts of AMF on the pathogen, such as competition for space or nutrients, or indirect, plant-mediated effects, can be implicated in AMF-mediated biocontrol. The latter can be further sub-divided into AMF’s influence on plant tolerance, plant defense induction, and altered plant exudation, all of which result in modifications in rhizosphere interactions (Kuila and Ghosh, 2022). The various mechanisms cannot be considered totally independent of one another, and biocontrol is most likely the outcome of a mix of mechanisms (Bell et al., 2016). Usually, AMF exhibit an antagonistic effect on plant-parasitic nematodes, and several studies have shown a significant reduction in nematodes when healthy mycorrhizal diversity present in the rhizosphere (Heinen et al., 2018; Gough et al., 2020; Poveda et al., 2020). In recent years, many investigations have been reported where AMF showed protective effects against plant parasitic nematodes (PPN) in various crop plants (Bagyaraj and Gautam Chawla, 2012; Vos et al., 2012, 2013; Alban et al., 2013; Bagyaraj, 2016; Sharma et al., 2021). However, only a few studies have been conducted so far to investigate the applications of AMF for the biocontrol of PPN in India (Bagyaraj and Gautam Chawla, 2012; Bagyaraj, 2018), and it is a need of the hour to explore these fungal symbionts for control of PPNs, especially RKN. Further there is no information available on AMF inducing key genes involved in plant defense and signaling for the biocontrol of RKN. With this in mind, the current study was conducted to investigate the role of selected AMF in the activation of plant defense responses against root-knot nematode *Meloidogyne graminicola* in rice (*Oryza sativa* L.) under direct seeded conditions.

## Materials and methods

### Source of media and reagents

Corn meal agar, potato dextrose agar and other media reagents were procured from HiMedia Private Limited (Mumbai, India). Other chemical reagents including analytical grade solvents and chemicals were purchased from E. Merck, Mumbai, India. Oligo nucleotide primers used in the present investigation were synthesized from Eurofins Genomics Services, Hyderabad, India. However, molecular grade chemicals were procured from BioRAD, India, Thermo Scientific, USA, and Bangalore GeNi, India.

### Source of AM fungi and inoculation techniques

The AMF fungal (*Funneliformis mosseae, R. fasciculatus*, and *R. intraradices*) inocula obtained from Centre For Natural Biological Resources and Community Development, Bangalore, India were maintained in pots with sterilized coco peat mixture (Ajay Kumar & Company, New Delhi, India) as the substrate and maize (*Zea mays* L., *cv*. Sujata) as the host according to Barea and Azcon-Aguilar (1983) with slight modifications. The AMF inoculum needed for the pot experiments, coco peat mixture was sterilized by autoclaving (at 121 °C for 20 min) twice at 24 h intervals. Thereafter, the coco peat mixture was kept as it is for the next 3 days to maintain the equilibrium of the cations and anions. The sterile coco peat mixture (1 kg) was filled in plastic pots (10 × 12 cm). To each pot, 10 g of AMF inoculum consisting of chopped AM-colonized root pieces along with the substrate containing spores and hyphal bits was added and mixed with the sterile coco peat. Ten healthy seeds of maize (*cv*. Sujata) were sown in each pot and watered as and when required. A total of 50 mL of Hoagland’s solution (without KH_2_PO_4_) (Hoagland and Arnon, 1950) was added to each pot at 15-days interval. After 75 days of sowing, the above-ground parts of the plants were cut and the roots separated from the substrate, cut into approximately 1 cm pieces, mixed with the substrate containing spores and hyphae, air-dried and used as the inoculum. The percent of AM root colonization and AM spore counts was calculated using the method of Gerdemann and Nicolson (1963) and Phillips and Hayman (1970) at 90 days of inoculation.

### Preparation of pathogen inoculum

*Meloidogyne graminicola* infested rice plants were collected from Research farm, Botanical Research Unit, Dhangain, Bikramganj, Rohtas; Agricultural Research Farm, Institute of Agricultural Sciences, Banaras Hindu University, Varanasi and farmers’ field, Rohtas districts of Southern Bihar, India and brought to the laboratory. Infested roots and galls were separated from plants using sharp scalpel and forceps. Infected roots and knots/galls were washed gently under running tap water. The 2nd stage juveniles (J_2_) were extracted following the protocols described by Singh et al. (2013a) with slight modifications. In brief, separated and cleaned root knots/galls were cut into small pieces with a sharp scalpel and placed on moist qualitative filter paper (Grade 4) mounted on cavity blocks containing sterilized distilled water with streptocycline (75 mg l^–1^) and cycloheximide (25 mg l^–1^). These cavity blocks were incubated at 27 ± 2°C for a week. A hemocytometer and nematode counting disc were used to count the number of second-stage juveniles (J2) (Singh, 2007) which were further used as nematode inoculum (2,500 J_2_ pot^–1^) (Singh et al., 2012c).

### Soil collection, preparation, and analysis

The experimental soil used in pot experiments was collected from the Research farm, Botanical Research Unit, Dhangain, Bikramganj, Bihar, India (coordinates: 25°21′2752″N 84°25′1975″E, Elevation 77 m) and brought to the laboratory. To eliminate the extra moisture, the soil was sieved (2 mm pore size) and air-dried. Vermiculture (obtained from ICAR-Indian Institute of Seed Sciences, Kushmaur, India) and river sand were blended into the soil in a 2:1:1 ratio (w/w). Required quantities of fertilizers for 3 kg soil were calculated and applied in liquid form using 0.314 g, 0.195 g, and 0.150 g of urea, diammonium phosphate and muriate of potash per pot, respectively. This represented the recommended dose of 120 kg ha^–1^N, 60 kg ha^–1^ P_2_O_5_, and 60 kg ha^–1^ K_2_O for direct seeded rice. Thereafter, experimental soil was sterilized by steam-sterilization (autoclaving) at 121°C for 30 min twice at 24 h intervals. After the second sterilization, the soil was kept as it is for the next 4−5 days to maintain the ionic balance/equilibrium in the cations and anions. Analysis of the physico-chemical characteristics of experimental soil was done using standard protocols described by Sparks et al. (2020).

### Planting materials and growth conditions

Susceptible and resistant breeding lines of rice (Pusa Basmati-1 and Jasmine 85, respectively) were obtained from the Department of Plant Breeding and Genetics, Veer Kunwar Singh College of Agriculture (Bihar Agricultural University-BAU, Sabour), Dumraon, Buxar, Bihar, India. Seeds were planted in pots (20 cm × 25 cm) under glass house conditions and each pot contained 3 kg of experimental soil with and without inoculation of AMF. The trials were conducted from July to September with a 13/−11 h light/dark photoperiod and relative humidity between 85 and 95 per cent. The annual average temperature in the region ranges from 27 to 32°C and the annual rainfall from 750 to 825 mm.

### Experimental set-up

Biocontrol efficacy of the AMF *F. mosseae, R. fasciculatus* and *R. intraradices* against root-knot diseases of rice were evaluated under glasshouse conditions at Veer Kunwar Singh College of Agriculture (Bihar Agricultural University-BAU, Sabour), Dumraon, Buxar, Bihar, India and ICAR-National Bureau of Agriculturally Important Microorganisms, Kushmaur, Maunath Bhanjan, India. The treatments were: *M. graminicola* (alone) (T_1_), *F. mosseae* + *M. graminicola* (T_2_), *R. fasciculatus* + *M. graminicola* (T_3_), *R. intraradices* + *M. graminicola* (T_4_), *F. mosseae* + *R. fasciculatus* + *R. intraradices* + *M. graminicola* (T_5_), and untreated Control (T_6_). In glasshouse trials, there were ten replications of each treatment using a completely randomized block design (CRBD).

To each pot, 10 g of AMF inoculum consisting of chopped AMF-colonized root pieces of maize, along with substrate containing about 75−100 AM spores g^–1^, was added. Rice seeds (inbred lines, PB-1 and Jasmine 85) were soaked in a brine solution (5% NaCl) for 10 min to remove the undersize and unhealthy seeds which floated on the surface. The healthy seeds, thus obtained, were surface-sterilized with sodium hypochlorite solution (NaOCl, 1% v/v) according to Singh et al. (2016). Ten seeds were sown in each pot with different treatments, later thinned to five when the third leaf appeared. After 30 days of sowing, second-stage juveniles (2,500 kg^–1^ of soil) were introduced near the root system. Nematode-free soil without AMF inoculum served as a control. The pots were randomly arranged according to CRBD in the glasshouse. Further, the positions of pots was re-randomized at weekly interval. To maintain the soil moisture, pots were watered on alternate days. The moisture was maintained at 60−70% of water holding capacity. The leachate came out from the hole was collected in saucer and reintroduced to the same pot. During the experimentation, temperature ranged from 25 to 32°C with 13/11 h photoperiod.

### Sampling and analysis

#### AM root colonization

After 30 and 45 days of sowing, the plants were up-rooted randomly from each treatment, washed in running tap water and brought to the laboratory. Five replicates per treatment were used. The mycorrhizal parameter, per cent root colonization was estimated following standard procedures (Phillips and Hayman, 1970; Giovannetti and Mosse, 1980).

#### Effect of AM inoculation on physio-biochemical parameters and antioxidant enzymes

The quantitative estimation of total chlorophyll content in the plant leaves was done according to Ferjani et al. (2003). The changes in the accumulation of total soluble sugar (TSS), total protein (TP), and total phenolic compounds (TPC) in the leaves and roots of plant primed with AMF fungal inocula and pre-challenged with the *M. graminicola* was estimated as per methods described by Sadasivam and Manickam (1996). Further, the activities and accumulation of phenylalanine ammonia-lyase (PAL), peroxidase (POx), ascorbate peroxidase (APx), polyphenol oxidase (PPO), superoxide dismutase (SOD), and catalase (CAT) were measured spectrophotometrically in the leaves and roots of AMF inoculated plants as per the protocols described by Thimmaiah (2012) at 30 days after pathogen inoculation (DAPI).

Further, AMF inoculation is known to affect the lignin content in plants challenged with biotic stresses. Hence, the rice root samples were collected 45 DAPI, washed thoroughly in running tap water and air-dried. The lignin content in the root samples was estimated spectrophotometrically as per the protocol defined by Yang et al. (2018).

#### Effect of AM inoculation on activation of defense-related pathways/cascades in roots

To see the effect of AM inoculation on the activation of defense-related pathways/cascades in the resistant and susceptible cultivar/inbred lines of rice pre-challenged with *M. graminicola*, quantitative real-time PCR analysis was carried out. In the present investigation, the expression of key genes involved in the induced systemic resistance, signaling processes, jasmonate biosynthesis, ethylene biosynthesis, BR biosynthesis, PR proteins, and lignin biosynthesis was studied in the rice plant under different treatments using qPCR. Sequences of 9 key genes regulating the phenylpropanoid cascade in rice were retrieved from National Centre for Biotechnology Information (NCBI) database. Gene-specific primers were designed for qPCR analyses and *in silico* validation of these primers was done. The 9 key genes analyzed were: Phenylalanine ammonia-lyase (*OsPAL*), Phenylalanine/tyrosine ammonia-lyase (*OsPAL/TAL*), 4-Coumarate-CoA ligase (*Os4-CL*), Cinnamoyl-CoA reductase (*OsCCR*), Cinnamyl-alcohol dehydrogenase (*OsCAD*), Peroxiredoxin 6 (*OsPOx*), Ferulate-5-hydroxylase (*OsF5H*), Caffeoyl-CoA O-methyltransferase (*OsCCoAOMT*), and Coniferyl-aldehyde dehydrogenase (*OsCALDH*). After 30 DAPI, plants from each treatment were harvested and brought to the laboratory in cool packs. The root samples were properly rinsed under running water to totally eliminate any traces of dirt. The clean root samples were quick-frozen in liquid nitrogen and ground and total RNA was extracted using a Total RNA isolation kit (Agilent, India) following the steps defined in the manufacturer’s protocols. There were three biological replicates. The cDNA was synthesized using a cDNA Synthesis Kit (BioRAD, India) following the manufacturer’s protocols. The quality and quantitative estimation of cDNA was determined using Nanodrop 2000c (Thermo Scientific, United States). The expression of these key genes was analyzed using gene-specific primers (Supplementary Table 1). The housekeeping gene *Actin* and *Glyceraldehyde 3-phosphate dehydrogenase* (GAPDH) was used as an endogenous standard to normalize the quantitative expression data. The qRT-PCR was performed according to Malviya et al. (2022) using BioRAD Real Time PCR System (MJ MiniOpticon, BioRAD). The relative transcript levels (fold change) were enumerated using the 2^–ΔΔCT^ method (Livak and Schmittgen, 2001) over the housekeeping gene *Actin* and GAPDH.

#### Effect of AMF inoculation on plant growth attributes and disease dynamics

To see how AMF inoculation affects plant growth characteristics in the rice plants pre-challenged with *M. graminicola*, plants were harvested from each treatment at 30 and 45 days after pathogen inoculation and taken to the workroom in cool packs. In order to completely remove the adhering dirt particles, the roots were extensively rinsed under running water. Root and shoot length and their fresh weight were determined.

Further, five plants from each treatment were sampled randomly to count the average records of root galls per root system, egg mass per root system, eggs per egg mass and J_2_ per root system in terms of numbers at 30 and 45 days after pathogen inoculation. The average number of J_2_ present in the root system was enumerated as per the method described by Bridge et al. (1981) with slight modifications. In brief, for enumeration of J_2_ population in the roots, root samples were stained for 30 min in a solution of boiling acid fuchsin (0.1 per cent w/v) in lactic acid, glycerol, and distilled water (1:1:1), macerated in distilled water and the number of J_2_ was counted under a light stereomicroscope.

### Statistical analysis

The laboratory experiments were laid out in a completely randomized design. The glasshouse experiments were laid out in a completely randomized block design (CRBD). Experiments were repeated twice. Data were subjected to analysis of variance (ANOVA) and compared with Duncan’s Multiple Range Test (DMRT) at *p* ≤ 0.05 using statistical package for Social Sciences Version 16.0 (SPSS Inc, 2007) programme. The root colonization data were transformed using an arcsine transformation and statistically analyzed using a CRBD using the SPSS version 16.0. Graphs were prepared using statistical software Origin (Version 9) and Microsoft Office Excel (2010).

## Results

### AM root colonization

To assess the effectiveness and potentiality of AMF inoculants to control the RKN infection and disease development, root colonization ability is an important attribute for any of the bioinoculants and it provides a clue for a commensal association between the two partners mediated through root exudates. Root colonization results indicated that rice roots were found to be colonized by all three strains tested at 30 and 45 DAPI. The per cent mycorrhizal root colonization was different for the three test species, *F. mosseae, R. fasciculatus* and *R. intraradices* in both the cultivars grown under glasshouse conditions and pre-challenged with *M. graminicola*. Among the three species studied, significantly higher root colonization was observed in the plants inoculated with *R. intraradices* at 30 and 45 DAPI (Table 1). However, per cent root colonization significantly increased when plants were inoculated with all the three AMF species together across the rice cultivars/inbred lines. Interestingly, Pusa Basmati-1 was found to be the best host as compared to Jasmine 85 (Table 1).

**TABLE 1:** Percent root colonization of *F. mosseae, R. fasciculatus*, and *R. intraradices* in rice cultivars at 30 and 45 days of sowing under glasshouse conditions.

Treatments	Pusa Basmati-1		Jasmine 85	
	**30 DAPI**	**45 DAPI**	**30 DAPI**	**45 DAPI**
T_1_- *M. graminicola*	00.00 (0.00)^e^	00.00 (0.00)^d^	00.00 (0.00)^d^	00.00 (0.00)^d^
T_2_- *M. graminicola* + *F. mosseae*	60.25 (50.91)^c^	78.10 (62.10)^b^	59.47 (50.46)^c^	68.60 (55.92)^c^
T_3_- *M. graminicola* + *R. fasciculatus*	55.74 (48.30)^d^	74.20 (59.47)^c^	62.40 (52.18)^c^	72.50 (58.37)^b^
T_4_- *M. graminicola* + *R. intraradices*	66.05 (54.36)^b^	80.26 (63.62)^b^	66.10 (54.39)^b^	74.10 (59.41)^b^
T_5_- *M. graminicola* + *F. mosseae* + *R. fasciculatus* + *R. intraradices*	75.47 (60.31)^a^	89.25 (70.86)^a^	70.50 (57.10)^a^	86.29 (68.27)^a^
T_6_- control (untreated)	00.00 (0.00)^e^	00.00 (0.00)^d^	00.00 (0.00)^d^	00.00 (0.00)^d^

Where, DAPI represents days after pathogen inoculation, Data are mean (*n* = 5). Data with different letters show significant difference in column data in randomized block design test at *p* < 0.05 under Duncan’s multiple range test. Figures in parenthesis indicate arcsine transformed averages.

### Effect of AM inoculation on physio-biochemical parameters and antioxidant enzymes

**FIGURE 1:**
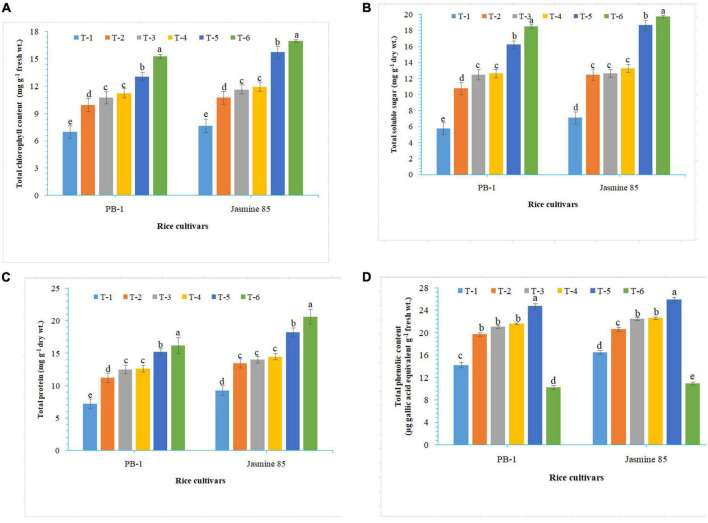
Effect of *F. mosseae, R. fasciculatus*, and *R. intraradices* inoculation on **(A)** total chlorophyll, **(B)** total soluble sugar, **(C)** total protein, and **(D)** total phenolic content in the shoot of susceptible inbred line, PB-1 and resistant inbred line, Jasmine 85 of rice pre-challenged with *M. graminicola* at 30 days of sowing under greenhouse conditions. Treatments were: T_1_- *M. graminicola*, T_2_- *M. graminicola* + *F. mosseae*, T_3_- *M. graminicola* + *R. fasciculatus*, T_4_- *M. graminicola* + *R. intraradices*, T_5_- *M. graminicola* + *F. mosseae* + *R. fasciculatus* + *R. intraradices*, and T_6_- Control (untreated). Column data are mean (*n* = 5) and vertical bar represents standard deviation. Data with different letters show significant difference in column data in randomized block design test at *p* < 0.05 under Duncan’s multiple range test.

**FIGURE 2:**
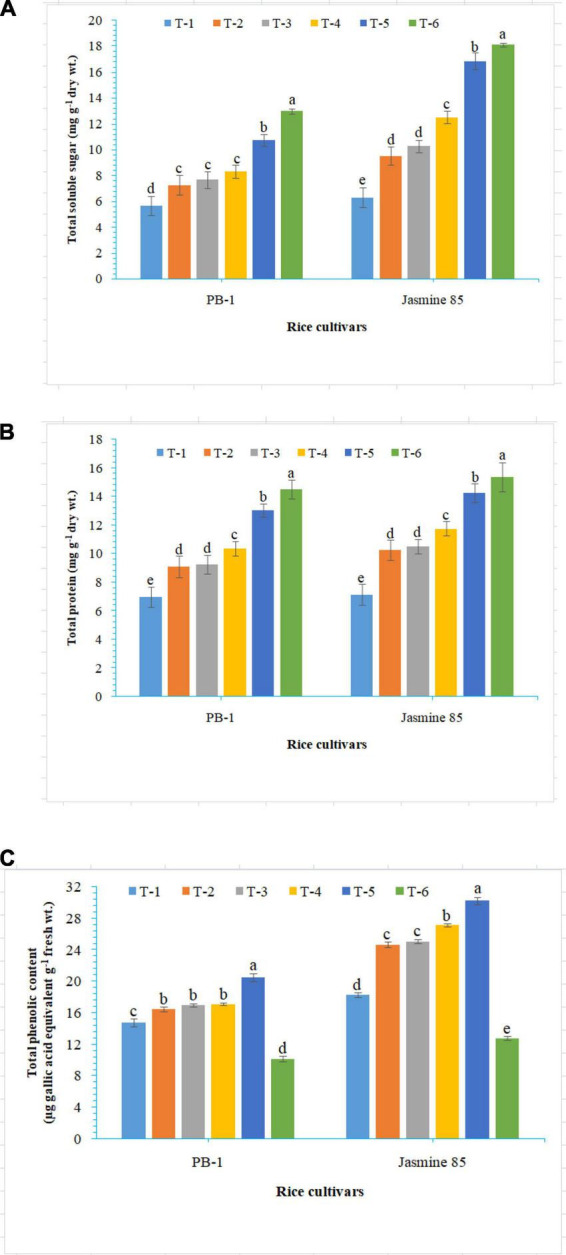
Effect of *F. mosseae, R. fasciculatus*, and *R. intraradices* inoculation on **(A)** total soluble sugar, **(B)** total protein, and **(C)** total phenolic content in the roots of susceptible inbred line, PB-1 and resistant inbred line, Jasmine 85 of rice pre-challenged with *M. graminicola* at 30 days of sowing under greenhouse conditions. Treatments were: T_1_- *M. graminicola*, T_2_- *M. graminicola* + *F. mosseae*, T_3_- *M. graminicola* + *R. fasciculatus*, T_4_- *M. graminicola* + *R. intraradices*, T_5_- *M. graminicola* + *F. mosseae* + *R. fasciculatus* + *R. intraradices*, and T_6_- Control (untreated). Column data are mean (*n* = 5) and vertical bar represents standard deviation. Data with different letters show significant difference in column data in randomized block design test at *p* < 0.05 under Duncan’s multiple range test.

To assess the mechanisms of tolerance in the susceptible and resistant inbred lines/cultivars of rice (Indica type) to RKN infection, accumulation and activities of defense-related biochemical and antioxidant enzymes, respectively, were measured spectrophotometrically at 30 DAPI. With respect to chlorophyll content, significantly higher content was reported in the absolute control (neither AMF inoculation nor pathogen challenge) at 30 DAPI (Figure 1A). Among the different treatments, plants inoculated with *F. mosseae, R. fasciculatus*, and *R. intraradices* in combination showed maximum chlorophyll content in both the inbred lines which were more close to the values reported in absolute control. However, *R. intraradices* inoculated plants performed better and compared to either *F. mosseae* or *R. fasciculatus* inoculated inbred lines pre-challenged with *M. graminicola*. The least amount of total chlorophyll was recorded in *M. graminicola* alone inoculated plants. In general, slightly higher chlorophyll content was reported in the resistant inbred line, Jasmine 85 as compared to PB-1 across the treatments (Figure 1A). In the content of TSS and TP, more or less a similar trend was observed in both susceptible and resistant inbred lines as recorded for total chlorophyll content in the absolute control and AM inoculated plants pre-challenged with *M. graminicola* in the glasshouse experiments (Figures 1B, C, respectively). In contrast, significantly higher accumulation of TPC was reported in the plants inoculated with *F. mosseae, R. fasciculatus* and *R. intraradices* in combination and pre-challenged by *M. graminicola* as compared to individually inoculated and pathogen alone inoculated plants (Figure 1D). However, the least TPC was reported in the absolute control. As reported for chlorophyll content, slightly higher TSS, TP, and TPC were recorded in the resistant inbred line, Jasmine 85 as compared to PB-1 across the treatments (Figures 1A–D). The TSS, TP, and TPC content was slightly lower in the roots of susceptible inbred line, PB-1 as compared to shoots across the treatments. However, the pattern was similar to shoot (Figures 2A–C). Further, the TSS and TP content in the roots of resistant line, Jasmine 85 was also lower as compared to shoot, while TPC was significantly higher in the roots as compared to shoot across the treatments (Figures 2A–C).

**FIGURE 3:**
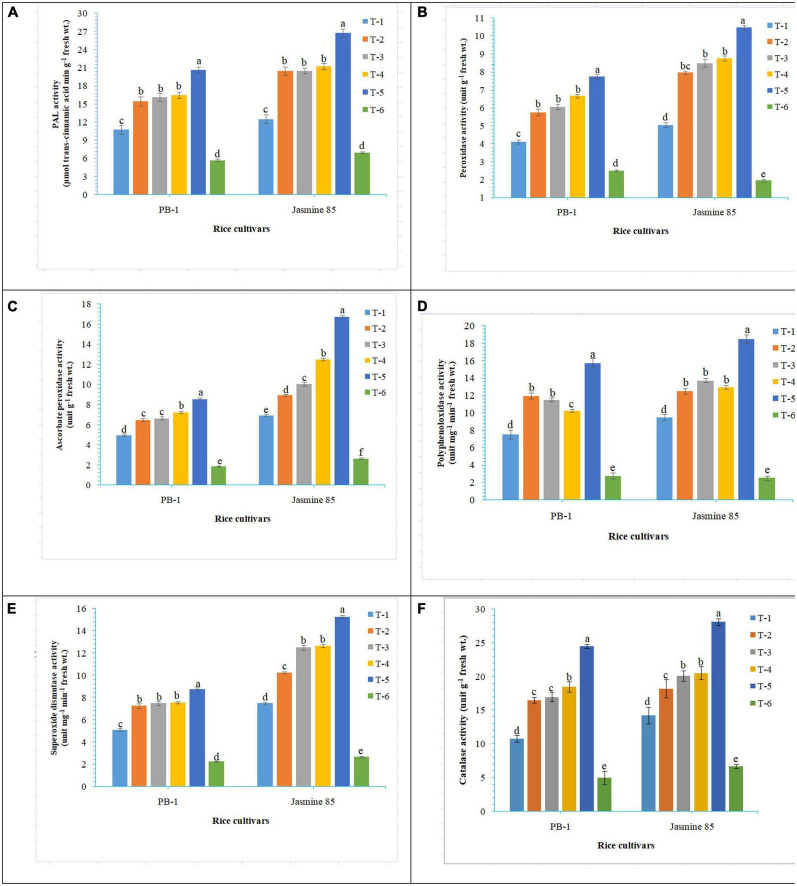
Effect of *F. mosseae, R. fasciculatus*, and *R. intraradices* inoculation on **(A)** PAL, **(B)** peroxidase, **(C)** ascorbate peroxidase, **(D)** polyphenol oxidase, **(E)** superoxide dismutase, and **(F)** catalase activity in the shoot of susceptible inbred line, PB-1 and resistant inbred line, Jasmine 85 of rice pre-challenged with *M. graminicola* at 30 days of sowing under greenhouse conditions. Treatments were: T_1_- *M. graminicola*, T_2_- *M. graminicola* + *F. mosseae*, T_3_- *M. graminicola* + *R. fasciculatus*, T_4_- *M. graminicola* + *R. intraradices*, T_5_- *M. graminicola* + *F. mosseae* + *R. fasciculatus* + *R. intraradices*, and T_6_- Control (untreated). Column data are mean (*n* = 5) and vertical bar represents standard deviation. Data with different letters show significant difference in column data in randomized block design test at *p* < 0.05 under Duncan’s multiple range test.

**FIGURE 4:**
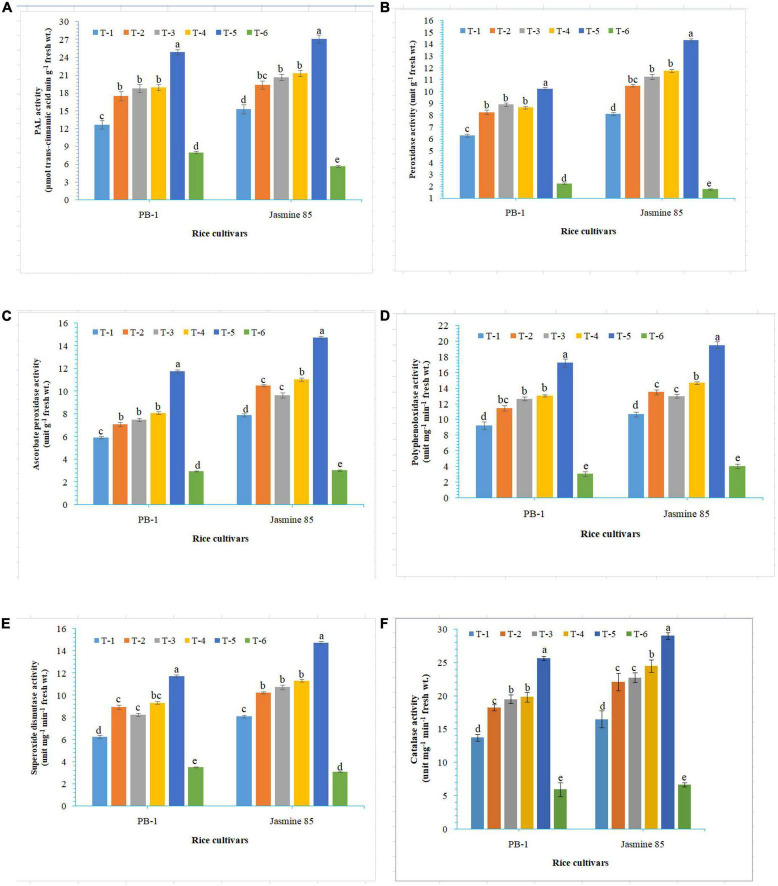
Effect of *F. mosseae, R. fasciculatus*, and *R. intraradices* inoculation on **(A)** PAL, **(B)** peroxidase, **(C)** ascorbate peroxidase, **(D)** polyphenol oxidase, **(E)** superoxide dismutase, and **(F)** catalase activity in the root of susceptible inbred line, PB-1 and resistant inbred line, Jasmine 85 of rice pre-challenged with *M. graminicola* at 30 days of sowing under greenhouse conditions. Treatments were: T_1_- *M. graminicola*, T_2_- *M. graminicola* + *F. mosseae*, T_3_- *M. graminicola* + *R. fasciculatus*, T_4_- *M. graminicola* + *R. intraradices*, T_5_- *M. graminicola* + *F. mosseae* + *R. fasciculatus* + *R. intraradices*, and T_6_- Control (untreated). Column data are mean (*n* = 5) and vertical bar represents standard deviation. Data with different letters show significant difference in column data in randomized block design test at *p* < 0.05 under Duncan’s multiple range test.

In order to gain an insight into the differential response of AM inoculated susceptible and resistant inbred lines upon *M. graminicola* infestation, the activity of antioxidant enzymes involved in the ROS-scavenging activities and plant defense was measured in the infested roots at 30 DAPI using UV vis spectrophotometer. Results revealed that significantly higher activity of PAL (26.25), POx (10.47), APx (16.76), PPO (18.50), SOD (15.26), and CAT (28.10) was recorded in the resistant inbred line, Jasmine 85 inoculated with consortia of *F. mosseae, R. fasciculatus* and *R. intraradices* and pre-challenged by *M. graminicola* as compared to individually inoculated and pathogen alone inoculated plants. The least activity of these antioxidant enzymes was observed in the absolute control (Figures 3A–F, respectively) as reported in case TPC. Interestingly, significantly less activity of these antioxidant enzymes was reported in the susceptible inbred line, PB-1 as compared to the resistant inbred line, Jasmine 85 across the treatments (Figures 3A–F). In contrast, the activity of PAL, POx, APx, PPO, SOD, and CAT was significantly higher in the roots of susceptible inbred line, PB-1 and resistant line, Jasmine 85 as compared to shoots across the treatments. However, the pattern was similar to shoot (Figures 4A–F).

### Effect of AM inoculation on activation of defense-related pathways/cascades

#### Expression of key genes involved in the MAPK pathway

**FIGURE 5:**
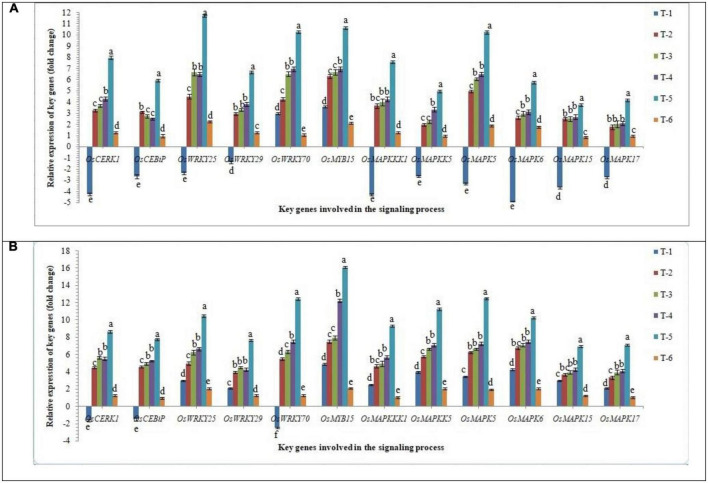
Effect of *F. mosseae, R. fasciculatus*, and *R. intraradices* inoculation on expression profile of key genes of involved in the signaling process in the **(A)** susceptible inbred line, PB-1 and **(B)** resistant inbred line, Jasmine 85 of rice pre-challenged with *M. graminicola* at 30 days of sowing under greenhouse conditions. Treatments were: T_1_- *M. graminicola*, T_2_- *M. graminicola* + *F. mosseae*, T_3_- *M. graminicola* + *R. fasciculatus*, T_4_- *M. graminicola* + *R. intraradices*, T_5_- *M. graminicola* + *F. mosseae* + *R. fasciculatus* + *R. intraradices*, and T_6_- Control (untreated). Column data are mean (*n* = 5) and vertical bar represents standard deviation. Data with different letters show significant difference in column data in randomized block design test at *p* < 0.05 under Duncan’s multiple range test.

In order to gain an insight into the differential response of AM inoculation in susceptible and resistant rice inbred lines/cultivars upon RKN infestation, the expression of key genes involved in the signaling process in root tissues was investigated in infested root using qRT-PCR at 30 DAPI. Mitogen-activated protein kinases (MAPK) involved as phosphorylation cascades in both pathogen-associated molecular pattern (PAMP)-triggered immunity (PTI) and effector-triggered immunity (ETI) and play a key role in signaling process during the early response to pathogens. Results indicated that RKN infestation drastically arrests the signaling process and in general, down-regulated the key genes involved in the signaling process in root tissues of susceptible inbred line, PB-1. Surprisingly, in the resistant inbred line, Jasmine 85, *OsCERK1*, *OSCEBiP*, and *OsWRKY70* genes were down-regulated slightly, while other genes tested returned to basal or even more expression levels in the infested roots of Jasmine 85 in comparison to the susceptible inbred line, PB-1 and untreated control plants (Figure 5). When we compared closely all the key genes taken into consideration up-regulated manifold upon inoculation of AMF fungi (2.50 to 11.74-fold). When consortia of *F. mosseae*, *R. fasciculatus*, and *R. intraradices* was inoculated to the roots of PB-1 pre-challenged by *M. graminicola* the mRNA levels of these genes were markedly increased. as compared to individually AMF inoculated, and pathogen alone inoculated plants (Figure 5).

On the other hand, transcript accumulation of *OsCERK1*, *OSCEBiP, OsWRKY25, OsWRKY29, OsWRKY70, OsMYB15, OsMAPKKK1*, *OsMAPKKK5*, *OsMAPK5*, *OsMAPK6*, *OsMAPK15*, and *OsMAPK17*, in general was significantly higher in resistant inbred line, Jasmine 85 as compared to susceptible line PB-1. The mRNA levels of these genes were considerably increased in the roots of Jasmine 85 upon inoculation of AMF consortia and pre-challenged with *M. graminicola* (3.5 to 16.10-fold) as compared to AMF and *M. graminicola* individually inoculated and untreated control plants (Figure 5). Conversely, a non-significant expression of *OsWRKYs, OsMYB, OsMAPKKKs*, and *OsMAPKs* was recorded in the root of untreated control plants (PB-1 and Jasmine 85 both) suggesting the key role of *MAPKs* and other genes in induced systemic resistance mechanisms of rice against *M. graminicola* infection (Figure 5).

#### Expression of key calcineurin B-like protein-interacting protein kinases genes

**FIGURE 6:**
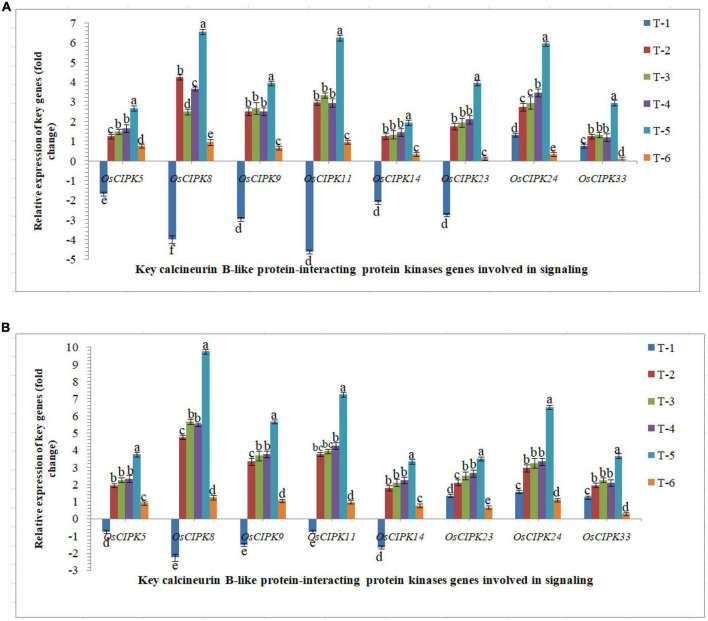
Effect of *F. mosseae, R. fasciculatus*, and *R. intraradices* inoculation on expression profile of key calcineurin B-like protein-interacting protein kinases genes involved in signaling in the **(A)** susceptible inbred line, PB-1 and **(B)** resistant inbred line, Jasmine 85 of rice pre-challenged with *M. graminicola* at 30 days of sowing under greenhouse conditions. Treatments were: T_1_- *M. graminicola*, T_2_- *M. graminicola* + *F. mosseae*, T_3_- *M. graminicola* + *R. fasciculatus*, T_4_- *M. graminicola* + *R. intraradices*, T_5_- *M. graminicola* + *F. mosseae* + *R. fasciculatus* + *R. intraradices*, and T_6_- Control (untreated). Column data are mean (*n* = 5) and vertical bar represents standard deviation. Data with different letters show significant difference in column data in randomized block design test at *p* < 0.05 under Duncan’s multiple range test.

The protein kinase, ‘calcineurin B-like protein-interacting protein kinase’ genes play important role in the signaling process upon pathogen-challenged and activated downstream biochemical pathways lead to plant defense. Differential expression was recorded in susceptible and resistant inbred lines upon RKN infection. The elevated transcriptional expression of the *CIPK* genes was verified by qRT-PCR analyses (Figure 6). The qRT-PCR results clearly indicated that nematode infestation suppresses the calcineurin B-like protein-interacting protein kinases-mediated signaling in the rice which was clearly evidenced from qRT-PCR analyses. Results clearly indicated that AM inoculation up-regulated the expression of *OsCIPK* genes, *OsCIPK5, OsCIPK8, OsCIPK9, OsCIPK11, OsCIPK14, OsCIPK23, OsCIPK24*, and *OsCIPK33* in both susceptible and resistant inbred lines across the treatments. Interestingly, like *OsMAPKKK, OsMAPKK, OsMAPKs, OsWRKY*s, and *OsMYB*, the higher expression was reported in the resistant line, Jasmine 85 inoculated with *F. mosseae, R. fasciculatus* and *R. intraradices* in combination and pre-challenged by *M. graminicola* as compared to individually inoculated and pathogen alone inoculated plants (Figure 6). A more or less similar trend was reported in the susceptible line, PB-1. However, the expression level (fold change) in PB-1 was significantly less as compared to Jasmine 85 across the treatments (Figure 6). In individual inoculation, the differences in the number of transcripts accumulated were not significant (*p* > 0.05) except for *OsCIPK8* in PB-1. The least expression was recorded in untreated controls where only basal level expression was recorded in both susceptible and resistant inbred lines (Figure 6).

#### Expression of key genes involved in the BR signaling and regulation

Despite CIPK and MAPK genes, genes involved in the BR signaling and regulation were induced by AMF inoculation in rice under pathogenic challenge. To examine the expression level of BR signaling and biosynthesis gene in response to *M. graminicola* infection, the key biosynthesis genes, *OsBRI1* (LOC_Os01g52050), *OsBAK1* (LOC_Os08g07760), *OsD2* (LOC_Os01g10040) and *OsD11* (LOC_Os04g39430) were down-regulated manifold in the susceptible line, PB-1 (−1.25 to −3.66 fold), whereas this value was slightly less in the resistant line, Jasmine 85 (−1.10 to −1.76 fold). However, AM inoculation up-regulated the *OsBRI1, OsBAK1, OsD2*, and *OsD11* genes in the root of rice plants pre-challenged with *M. graminicola*. Results revealed that significantly higher expression of *OsBRI1* (4.19-fold), *OsBAK1* (6.96-fold), *OsD2* (3.90-fold), and *OsD11* (2.66-fold) genes was recorded in the rice cultivar, PB-1 inoculated with consortia of *F. mosseae, R. fasciculatus* and *R. intraradices* and pre-challenged by *M. graminicola* as compared to individually inoculated and pathogen alone inoculated plants (Supplementary Figure 1). A more or less similar trend was recorded in the resistant cultivar/inbred line, Jasmine 85. Interestingly, in the resistant line, the effect of RKN on the expression of BR biosynthesis genes was significantly less (Supplementary Figure 1). It was also observed that basal level expression of BR biosynthesis genes always recorded in the susceptible and resistant plants which clearly indicated that these genes were not only involved in the plant defense but also played another role in plant development and reproduction (Supplementary Figure 1).

#### Expression of key genes involved in the jasmonate biosynthesis

During nematodes and vertebrates’ invasion, jasmonate-dependent pathways played important role in the plant defense and coping with the negative impact of these invaders. Among different key genes of jasmonate biosynthesis and signaling, *OsAOS2* (a key enzyme in JA biosynthesis), *OsJMT1* (converts JA to volatile MeJA), and *OsJAMYB* (JA-inducible MYB transcription factor) were used as the key marker genes to investigate the JA-dependent responses in plants including rice. Results of the present investigation clearly indicated that RKN infestation down-regulated the expression of *OsAOS2* (−1.66 fold) and *OsJMT1* (−1.96 fold) in the susceptible line, PB-1. While AM inoculation alone or in combination significantly up-regulated the genes involved in the JA biosynthesis and downstream signaling in both susceptible and resistant lines under the pathogenic stress of *M. graminicola* (Supplementary Figure 2). It was clearly observed that significantly higher expression of *OsAOS2, OsJMT1*, and *OsJAMYB* genes was recorded in the rice cultivars, PB-1 and Jasmine 85 inoculated with *F. mosseae, R. fasciculatus* and *R. intraradices* in combination and pre-challenged by *M. graminicola* as compared to individually inoculated and pathogen alone inoculated plants (Supplementary Figure 2). The least expression was reported in untreated control plants.

#### Expression of key genes involved in the ethylene biosynthesis

Ethylene is the key phytohormone playing an important role in signaling, plant defense and plant growth as a whole. Ethylene, at lower concentrations induced IAA-mediated root development and downstream regulation of plant defense under pathogenic stresses. In general, AM-mediated induction of ethylene biosynthesis and downstream signaling in rice has not been reported so far. ET biosynthesis and signaling genes were either down-regulated or up-regulated differentially in the roots of the susceptible and resistant plant at 30 DAPI. However, in the present investigation, AM inoculation up-regulated key genes involved in ethylene biosynthesis and signaling. Among them, *OsACS1, OsACO7* (two major catalytic enzymes involved in the biosynthesis of ET from methionine), *OsEIN2* (central signal transducer in ET signaling pathway), and *OsERF1* (ET-inducible gene) are the most important and were used as the marker genes to demonstrate the ET-related responses. Results of the present investigation clearly indicated that *M. graminicola* attenuated the expression of these genes in the infected root of the susceptible line, PB-1 (−1.25 to −4.67 fold) as compared to the resistant inbred line, Jasmine 85 (−1.25 to −1.66 fold). Inoculation of AM fungi, *F. mosseae, R. fasciculatus*, and *R. intraradices* individually or in combination up-regulated and mRNA levels of *OsACS1* and *OsACO7* were increased manifold (∼2-fold) as compared to *OsEIN2*, and *OsERF1* in the infected root of PB-1 at 30 DAPI. A near baseline expression of *OsACS1, OsACO7, OsEIN2*, and *OsERF1* was documented in the root of uninoculated control line PB-1 at 30 DAPI (Supplementary Figure 3). On the contrary, a strong induction of *OsACO7* was observed in the roots of Jasmine 85 inoculated with *F. mosseae, R. fasciculatus* and *R. intraradices* individually or in combination at 30 DAPI. However, the transcripts of *OsACS1, OsEIN2*, and *OsERF1* were significantly induced in the roots of Jasmine 85 inoculated with *F. mosseae, R. fasciculatus* and *R. intraradices* individually or in combination 5 at 30 DAPI (Supplementary Figure 3). Collectively, a consistent overexpression of ethylene-responsive genes throughout the course of nematode infection in the resistant and susceptible plants suggests a positive correlation between ET-inducible gene expression in rice co-inoculated with AM fungi and overall defense to *M. graminicola*.

#### Expression of genes encoding pathogenesis-related proteins

**FIGURE 7:**
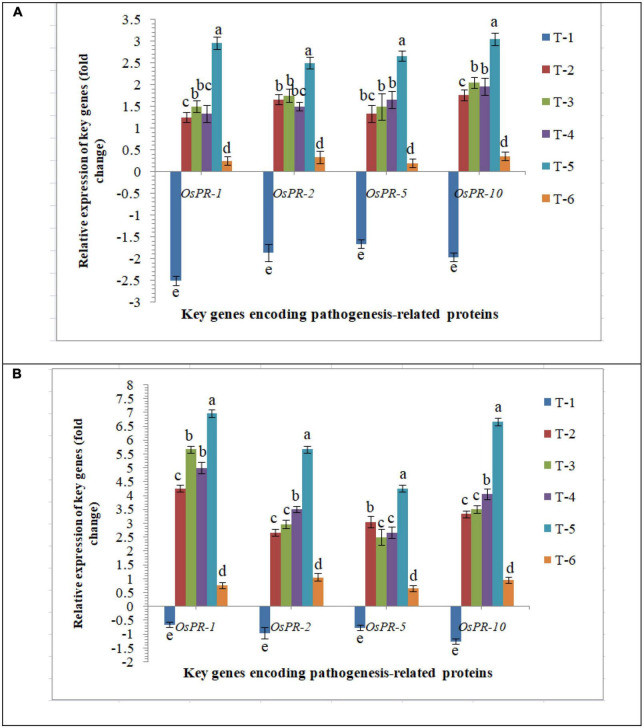
Effect of *F. mosseae, R. fasciculatus*, and *R. intraradices* inoculation on expression profile of genes encoding pathogenesis-related proteins in the **(A)** susceptible inbred line, PB-1 and **(B)** resistant inbred line, Jasmine 85 of rice pre-challenged with *M. graminicola* at 30 days of sowing under greenhouse conditions. Treatments were: T_1_- *M. graminicola*, T_2_- *M. graminicola* + *F. mosseae*, T_3_- *M. graminicola* + *R. fasciculatus*, T_4_- *M. graminicola* + *R. intraradices*, T_5_- *M. graminicola* + *F. mosseae* + *R. fasciculatus* + *R. intraradices*, and T_6_- Control (untreated). Column data are mean (*n* = 5) and vertical bar represents standard deviation. Data with different letters show significant difference in column data in randomized block design test at *p* < 0.05 under Duncan’s multiple range test.

General defense responses in rice inoculated with *F. mosseae, R. fasciculatus* and *R. intraradices* individually or in combination upon *M. graminicola* infection were investigated. To elucidate the molecular mechanisms sustaining the general defense response of rice triggered upon AMF inoculation and nematode infection, the differential expression of PR genes, *OsPR1, OsPR2, OsPR5*, and *OsPR10* was evaluated. Despite differential expression at 30 DAPI mRNA levels of *OsPR1, OsPR2, OsPR5*, and *OsPR10* were attenuated in the infected root of PB-1 inoculated with *F. mosseae, R. fasciculatus* and *R. intraradices* individually or in combination. An increased and approximate consistent transcript abundance of *OsPR1, OsPR2, OsPR5*, and *OsPR10* was recorded in the roots of susceptible inbred line, PB-1 at 30 DAPI (Figure 7). However, the least expression was reported in untreated control plants. The strong and differential up-regulation of *OsPR1, OsPR2, OsPR5*, and *OsPR10* genes found in the roots of resistant line, Jasmine 85 inoculated with *F. mosseae, R. fasciculatus* and *R. intraradices* individually or in combination at 30 DAPI confirms the defense-inducing capabilities of PR genes in rice roots in response to RKN attack. However, the strongest up-regulation of *OsPR1* was recorded in Jasmine 85 at 30 DAPI. Conversely, expression of *OsPR1, OsPR2, OsPR5*, and *OsPR10* was markedly down-regulated in the infected root of Jasmine 85 as compared to AM-inoculated plants (Figure 7).

#### Expression of key genes involved in the phenylpropanoid pathway

**FIGURE 8:**
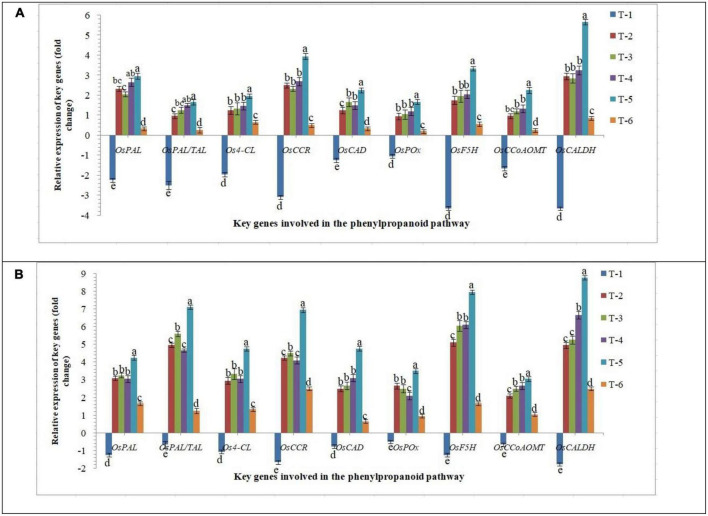
Effect of *F. mosseae, R. fasciculatus*, and *R. intraradices* inoculation on expression profile of key genes involved in the phenylpropanoid pathway in the **(A)** susceptible inbred line, PB-1 and **(B)** resistant inbred line, Jasmine 85 of rice pre-challenged with *M. graminicola* at 30 days of sowing under greenhouse conditions. Treatments were: T_1_- *M. graminicola*, T_2_- *M. graminicola* + *F. mosseae*, T_3_- *M. graminicola* + *R. fasciculatus*, T_4_- *M. graminicola* + *R. intraradices*, T_5_- *M. graminicola* + *F. mosseae* + *R. fasciculatus* + *R. intraradices*, and T_6_- Control (untreated). Column data are mean (*n* = 5) and vertical bar represents standard deviation. Data with different letters show significant difference in column data in randomized block design test at *p* < 0.05 under Duncan’s multiple range test.

Phenylpropanoid is the key pathway induced in plants under biotic and abiotic stresses and modulates the downstream defense/tolerance mechanisms in a different manner. Similar to other signaling mechanisms discussed above, differential expression of crucial phenylpropanoid pathway-related genes was reported in the roots of susceptible and resistant inbred lines inoculated with AM fungi upon RKN infection. Nine marker genes, *OsPAL, OsPAL/TAL, Os4-CL, OsCCR, OsCAD, OsPOx, OsF5H, OsCCoAOMT*, and *OsCALDH* were used as the marker genes to demonstrate the phenylpropanoid-related responses. While in roots of PB-1, transcripts of *OsPAL, OsPAL/TAL, Os4-CL, OsCCR, OsCAD, OsPOx, OsF5H, OsCCoAOMT*, and *OsCALDH* were either unaltered or attenuated under RKN infection at 30 DAPI (Figure 8). The transcripts of *OsPAL, OsPAL/TAL, Os4-CL, OsCCR, OsCAD, OsPOx, OsF5H, OsCCoAOMT*, and *OsCALDH* were strongly up-regulated in Jasmine 85 in contrast to significantly less expression in PB-1 inoculated with *F. mosseae, R. fasciculatus* and *R. intraradices* individually or in combination at 30 DAPI (Figure 8). Conversely, a non-significant expression of these genes was documented in the RKN-infected root of PB-1 and Jasmine 85 inoculated with *F. mosseae, R. fasciculatus*, and *R. intraradices* individually at 30 DAPI, suggesting the equivocal role of these genes in induced systemic defense of rice against RKN (Figure 8). In concordance with these findings, it appeared that key genes involved in the phenylpropanoid pathway had a major positive effect in activating the systemic defense of susceptible and resistant inbred lines of rice in response to RKN attack.

#### Expression of key genes involved in the lignin and callose biosynthesis

**FIGURE 9:**
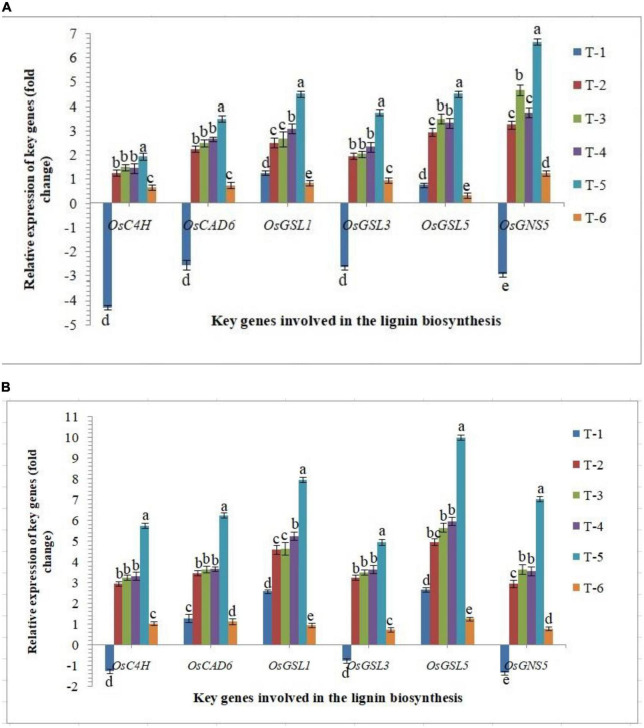
Effect of *F. mosseae, R. fasciculatus*, and *R. intraradices* inoculation on expression profile of key genes involved in the lignin and callose biosynthesis in the **(A)** susceptible inbred line, PB-1 and **(B)** resistant inbred line, Jasmine 85 of rice pre-challenged with *M. graminicola* at 30 days of sowing under greenhouse conditions. Treatments were: T_1_- *M. graminicola*, T_2_- *M. graminicola* + *F. mosseae*, T_3_- *M. graminicola* + *R. fasciculatus*, T_4_- *M. graminicola* + *R. intraradices*, T_5_- *M. graminicola* + *F. mosseae* + *R. fasciculatus* + *R. intraradices*, and T_6_- Control (untreated). Column data are mean (*n* = 5) and vertical bar represents standard deviation. Data with different letters show significant difference in column data in randomized block design test at *p* < 0.05 under Duncan’s multiple range test.

Lignin and callose deposition in plant roots play a crucial role in plant defense against biotic stresses. Significantly higher accumulation reinforces the plant tissues by conferring mechanical strength to cell walls against invading pathogens including RKN. In the present investigation, the expression of key genes involved in the lignin and callose biosynthesis was studied in the roots of susceptible and resistant inbred lines, PB-1 and Jasmine 85, respectively, inoculated with AMF. As revealed in Figure 9, the expression of *OsC4H, OsCAD6* (two lignin biosynthesis genes), *OsGSL1* (callose synthase genes) and *OsGNS5* (callose hydrolyzing gene) was down-regulated significantly in the roots of PB-1 challenged with *M. graminicola*. However, inoculation of *F. mosseae, R. fasciculatus* and *R. intraradices* individually or in combination strongly up-regulated the expression of *OsC4H, OsCAD6, OsGSL1, OsGSL3, OsGSL5* and *OsGNS5* in the roots of susceptible and resistant inbred lines (Figure 9). In agreement with these results, significantly higher expression of these genes was observed in the RKN-infected root of resistant plants compared to susceptible ones at 30 DAPI (Figure 9). These findings supported the notion that AM fungi modulated the expression of key genes involved in lignin and callose deposition and might play the pivotal role in inhibiting nematode penetration and consequently, the delayed development and reproduction of RKN occured in the resistant inbred line, Jasmine 85. This finding is attributable to the possible role of lignin and callose-related genes in rice basal defense against nematodes (Figure 9).

### Effect of AM inoculation on lignin content

**FIGURE 10:**
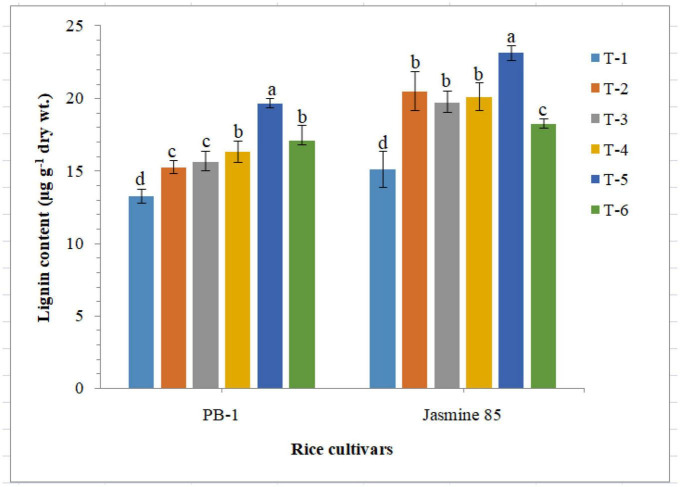
Effect of *F. mosseae, R. fasciculatus*, and *R. intraradices* inoculation on lignin content in the susceptible inbred line, PB-1 and resistant inbred line, Jasmine 85 of rice pre-challenged with *M. graminicola* at 45 days of sowing under greenhouse conditions. Treatments were: T_1_- *M. graminicola*, T_2_- *M. graminicola* + *F. mosseae*, T_3_- *M. graminicola* + *R. fasciculatus*, T_4_- *M. graminicola* + *R. intraradices*, T_5_- *M. graminicola* + *F. mosseae* + *R. fasciculatus* + *R. intraradices*, and T_6_- Control (untreated). Column data are mean (*n* = 5) and vertical bar represents standard deviation. Data with different letters show significant difference in column data in randomized block design test at *p* < 0.05 under Duncan’s multiple range test.

Quantitative analysis revealed that inoculation of *F. mosseae, R. fasciculatus* and *R. intraradices* individually or in combination enriched lignin synthesis and accumulation in susceptible and resistant inbred lines, PB-1 and Jasmine 85. Results of the present investigation clearly indicated that RKN infection hampered the lignin synthesis and accumulation in a significant way (Figure 10). As compared to other treatments, significantly higher accumulation of lignin was reported in the plants inoculated with consortia of the three AMF. However, the least lignin was recorded in the roots of PB-1 followed by Jasmine 85 pre-challenged with *M. graminicola* (Figure 10).

### Effect of AMF inoculation on disease dynamics

**FIGURE 11:**
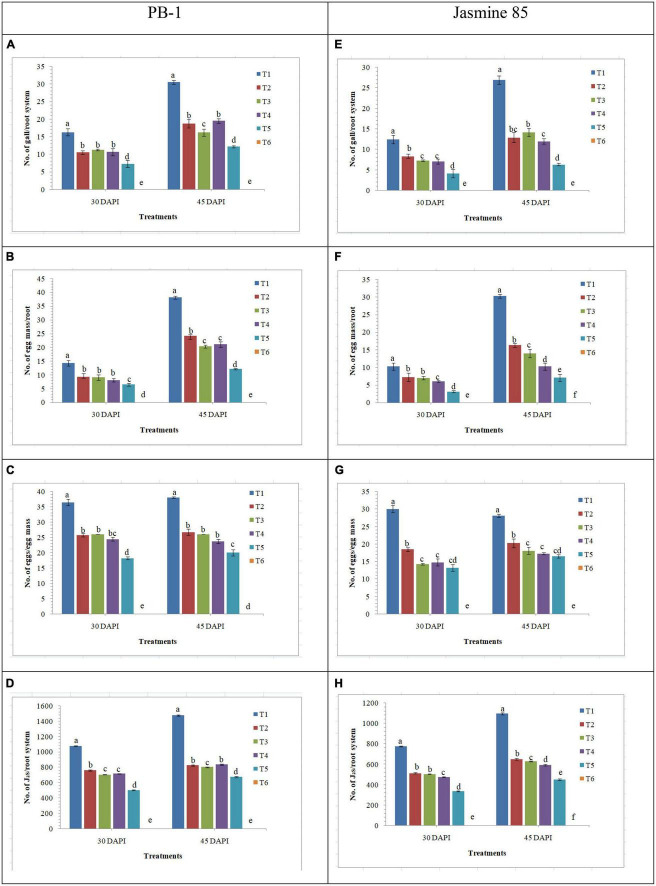
Effect of *F. mosseae, R. fasciculatus*, and *R. intraradices* inoculation on root gall development under biotic stress of *M. graminicola* in the susceptible (PB-1) **(A–D)** and resistant (Jasmine-85) **(E–H)** inbred lines of rice at 30 and 45 days of pathogen inoculation under net house conditions. Treatments were: T_1_- *M. graminicola*, T_2_- *M. graminicola* + *F. mosseae*, T_3_- *M. graminicola* + *R. fasciculatus*, T_4_- *M. graminicola* + *R. intraradices*, T_5_- *M. graminicola* + *F. mosseae* + *R. fasciculatus* + *R. intraradices*, and T_6_- Control (untreated). Column data are mean (*n* = 5) and vertical bar represents standard deviation. Data with different letters show significant difference in column data in randomized block design test at *p* < 0.05 under Duncan’s multiple range test.

The effect of AMF inoculation on the development of root gall, production of egg mass and enumeration of J_2_s in the root system was investigated in the susceptible and resistant inbred lines of rice pre-challenged with *M. graminicola.* Significantly higher number of galls was recorded in the inbred line PB-1 inoculated with *M. graminicola* alone (16.25 galls/root system) as compared to the *F. mosseae, R. fasciculatus*, and *R. intraradices* inoculated plants (10.47, 11.25, and 10.66 galls/root system, respectively). However, the least number of galls was reported in the roots of PB-1 inoculated with consortia of the three AMF (7.25 galls/root system) at 30 DAPI (Figure 11A). Further results revealed that the total number of egg mass per root system was higher in the roots of PB-1 infected with *M. graminicola* (14.25 egg mass/root system) alone than in plants inoculated with *F. mosseae, R. fasciculatus* and *R. intraradices* individually (9.27, 9.05, and 8.10 egg mass/root system, respectively) or in combination (6.50 egg mass/root system) under pathogenic stress of *M. graminicola* (Figure 11B). Results revealed that the number of eggs per egg mass varied in different treatments. The maximum of eggs per egg mass was recorded in the roots of PB-1 pre-challenged with *M. graminicola* (36.50) at 30 DAPI (Figure 11C). However, the sum of eggs per egg mass decreased significantly in the root of PB-1 inoculated with *F. mosseae, R. fasciculatus*, and *R. intraradices* individually (25.75, 26.10, and 24.47 eggs/egg mass, respectively) or in combination (18.25 eggs/egg mass). At 30 DAPI, J_2_ populations were significantly higher in the roots of PB-1 infected with *M. graminicola* alone (1076.25 J_2_/root system) as compared to *F. mosseae, R. fasciculatus*, and *R. intraradices* inoculation individually (759.25, 702.10, and 715.21 J_2_/root system, respectively) or in combination (501.25 J_2_/root system) inoculated plants under pathogenic stress of *M. graminicola* (Figure 11D). After 45 DAPI, 2nd observation on the average number of galls per plant, egg mass per root system, eggs per egg mass and J_2_/root system was taken. Results revealed that the average number of galls per plant increased by 1.50 to 2.00 times, egg mass per root system by 2.00 to 7.75-fold, and J_2_/root system by 1.25 to 1.50-fold in the roots of PB-1 under different treatments. However, eggs per egg mass did not change significantly at 30 and 45 DAPI (Figures 11A–D). Further analysis showed that the average number of galls per plant, egg mass per root system, eggs per egg mass and J_2_/root system were significantly lower in the roots of the resistant inbred line, Jasmine 85 as compared to the susceptible line, PB-1 at 30 and 45 DAPI (Figures 11E–H).

### Effect of AM inoculation on plant growth attributes

To assess how AM inoculation affects plant growth characteristics, glasshouse experiments were conducted in susceptible and resistant inbred lines of rice. The plant growth attributes, i.e., shoot and root length and fresh weight of shoot and root increased significantly in plants inoculated with the three AMF individually or in combination in both the susceptible and resistant inbred lines as compared to pathogen alone treated plants. In general, plant growth attributes increased 1.50 to 2.00 times in the plants inoculated with the consortia of three AMF as compared to pathogen alone treated plants at 30 and 45 DAPI (Table 2).

**TABLE 2:** Effect of *F. mosseae, R. fasciculatus*, and *R. intraradices* inoculation on plant growth attributes under biotic stress of *M. graminicola* in rice at 30 and 45 days of pathogen inoculation under net house conditions.

Treatments	Shoot length (cm)		Root length (cm)		Shoot fresh wt. (mg)		Root fresh wt. (mg)	
	**30 DAPI**	**45 DAPI**	**30 DAPI**	**45 DAPI**	**30 DAPI**	**45 DAPI**	**30 DAPI**	**45 DAPI**
**Rice cultivar: Pusa Basmati-1**								
T_1_- *M. graminicola*	28.25^d^	36.20^d^	16.50^e^	20.89^e^	130.33^d^	145.20^d^	21.50^c^	29.47^d^
T_2_- *M. graminicola* + *F. mosseae*	43.46^c^	55.25^c^	22.47^d^	30.25^d^	166.25^c^	190.50^c^	24.46^b^	30.75^d^
T_3_- *M. graminicola* + *R. fasciculatus*	40.75^b^	59.21^b^	25.50^c^	34.45^c^	176.50^b^	199.25^b^	24.05^b^	32.10^c^
T_4_- *M. graminicola* + *R. intraradices*	39.50^b^	60.47^b^	27.10^b^	38.50^b^	175.10^b^	202.15^b^	23.50^b^	32.50^c^
T_5_- *M. graminicola* + *F. mosseae* + *R. fasciculatus* + *R. intraradices*	46.91^a^	66.25^a^	31.50^a^	41.50^a^	189.50^a^	226.75^a^	26.25^a^	34.15^b^
T_6_- control (untreated)	47.50^a^	60.50^b^	30.45^a^	42.75^a^	190.25^a^	225.20^a^	25.10^ab^	36.27^a^
**Rice cultivar: Jasmine 85**								
T_1_- *M. graminicola*	26.46^e^	32.10^e^	18.50^e^	25.47 ^d^	135.20^e^	150.25^e^	22.45^e^	31.75^b^
T_2_- *M. graminicola* + *F. mosseae*	34.25^cd^	46.25^d^	25.25^d^	34.29^c^	175.50^d^	210.20^d^	26.29^d^	29.10^c^
T_3_- *M. graminicola* + *R. fasciculatus*	35.75^c^	49.11^c^	28.75^c^	38.50^b^	181.25^c^	215.33^c^	26.75^d^	28.75^c^
T_4_- *M. graminicola* + *R. intraradices*	36.05^c^	50.25^c^	28.07^c^	40.10^b^	180.75^c^	216.50^c^	24.10^c^	29.35^c^
T_5_- *M. graminicola* + *F. mosseae* + *R. fasciculatus* + *R. intraradices*	38.10^b^	55.33^b^	34.20^a^	45.50^a^	190.10^b^	225.15^b^	30.50^b^	32.12^b^
T_6_- control (untreated)	40.50^a^	56.05^a^	32.40^b^	46.05^a^	195.50^a^	230.33^a^	31.05^a^	33.90^a^

Where, DAPI represents days after pathogen inoculation, Data are mean (*n* = 10). Data with different letters show significant difference in column data in randomized block design test at *p* < 0.05 under Duncan’s multiple range test.

## Discussion

The current day emphasis is on sustainable agriculture, which uses less of chemical inputs having adverse effect on soil health, and environment. The arbuscular mycorrhizal fungi (AMF) and other microbial inoculants play an important role in sustainable agriculture (Smith and Read, 2008; Akhmetzhanova et al., 2012; Babikova et al., 2013; Kammadavil Sahodaran et al., 2019; Lakshmipathy et al., 2019). There are several reports on AMF-soil-borne plant pathogen interactions (Bagyaraj, 2016; Singh A. et al., 2019; Singh U. B. et al., 2019). Most of the studies on AMF-root pathogens suggest that AMF decreased or mitigated the disease severity. Consistent reduction of disease symptoms has been described for fungal, bacterial and nematode pathogens. Studies conducted so far suggest that the mechanisms of suppression may be due to morphological, physiological and biological alterations in the host (Ijdo et al., 2011; Nahar et al., 2011; Khan et al., 2012; Frac et al., 2018). It includes thickening of the cell walls through lignification preventing penetration of pathogens and activation of specific defense mechanisms such as production of phytoalexins, chitinases, pathogenesis related proteins and enhanced antagonists in the mycorrhizosphere (Nahar et al., 2012; Bagyaraj, 2018). In the present investigation, inoculation of AM fungi, *F. mosseae, R. fasciculatus*, and *R. intraradices* individually and in combination under RKN challenge modulated physio-biochemical cascades in rice led to downstream signaling. Application of AM fungi not only modulates physio-biochemical pathways but also reduced the infestation, colonization, and invasion of RKN, *M. graminicola*. Results indicated that *F. mosseae, R. fasciculatus*, and *R. intraradices* when inoculated individually or in combination, colonized the rice roots to a greater extent (55.74 to 89.25%) thereby occupy maximum area of the root along with increased hyphal network in soil and thereby helping in enhanced plant nutrition (Brundrett, 2002, 2009; Johansson et al., 2004). Enhanced AMF colonization increased lateral root formation (secondary and tertiary rooting) and modulates the root morphology of rice which is beneficial for plant growth and development (Kyndt et al., 2012a; van der Heijden et al., 2015). During the course of screening of rice germplasm, PB-1 was found to be the most susceptible cultivar and Jasmine 85 the most resistant cultivar to *M. graminicola* infection at 30 DAPI. Hence, the rice-*M. graminicola* pathosystem with the susceptible and resistant inbred line was taken as a model system to elucidate the interactions between RKN and the host (Kyndt et al., 2012a,b; Nguyễn et al., 2014). In plants, immune system responses are in general regulated by effector-triggered immunity (ETI) and pattern-triggered immunity (PTI). PTI is generally activated when plant perceive microbial structures, in general, referred to as pathogen-associated molecular patterns (PAMPs), *via* the transmembrane pattern recognition receptors (PRRs) (Lee et al., 2004; Gheysen and Jones, 2006; Hewezi et al., 2010). However, ETI is activated when plants recognize specific effector molecules produced by pathogens *via* intracellular nucleotide-binding leucine-rich repeat (NLR) receptors, called resistance (R) proteins (Sanz-Alferez et al., 2008; Hamamouch et al., 2011;

Molinari et al., 2014) and thereby effectively up-regulate the defense mechanisms inside plant cells in response to pathogen infection and invasion (Harman et al., 2004; Singh U. B. et al., 2019; Malviya et al., 2022).

Further, mitogen-activated protein (MAP) kinase (MAPK) signaling pathways play a crucial role in plant defense, hypersensitive response (HR) reaction, immune responses, and oxidative burst to pathogen attack (Zhang and Klessig, 2001; Bari and Jones, 2009; Pieterse et al., 2009). Moreover, the microbe-mediated activation of MAPKs and downstream signaling networks in plants has not been completely defined. In general, 74 MAPKKK, 8 MAPKK, and 17 MAPK genes have been reported so far in the rice genome (Hamel et al., 2006; Reyna and Yang, 2006; Rao et al., 2010; Yang et al., 2015). They are playing a different role in signaling cascades underlying the physiological and cellular responses in rice (Tamogami et al., 1997; Schweizer et al., 1998; van Loon et al., 2006; Singh et al., 2012a). In the present investigation, RKN infection down-regulated the expression of key genes involved in the MAPK-mediated signaling pathways in the susceptible cultivar, PB-1 (−1.39 to −4.29 fold) as compared to the resistant inbred line, Jasmine 85. Further inoculation of AM fungi, *F. mosseae, R. fasciculatus* and *R. intraradices* individually or in combination up-regulated the expression of these genes in both susceptible and resistant inbred lines, PB-1, and Jasmine 85, respectively, pre-challenged with *M. graminicola*. This advocates a key role for MAPK signaling in rice response to RKN infection (Kumari et al., 2016; Zhou et al., 2020). To the best of our knowledge and the literatures available, this is the first report on the role of *F. mosseae, R. fasciculatus*, and *R. intraradices* in the activation of MAPK signaling in rice under pathogenic challenge of RKN *M. graminicola* in rice, which is further confirmed by the enhanced systemic resistance in susceptible and resistant inbred lines of rice (Pozo and Azcon-Aguilar, 2007). According to Rodriguez et al. (2009), “Plant MAPK cascades proceed through three central kinases: MAPK kinase kinase (MAPKKK); MAPK kinase (MAPKK), also known as MAPK and ERK (extracellular signal regulated kinase) kinase (MEK); and MAP kinase (MAPK or MPK).” In the present investigation, *OsCERK1*, *OSCEBiP, OsWRKY25, OsWRKY29, OsWRKY70, OsMYB15, OsMAPKKK1*, *OsMAPKKK5*, *OsMAPK5*, *OsMAPK6*, *OsMAPK15*, and *OsMAPK17* were highly expressed in the rice plant primed with the selected strains of AM fungi, *F. mosseae, R. fasciculatus*, and *R. intraradices* individually or in combination. The MAPKs further activated *OsWRKYs*, and *OsMYB* transcription factors cause HR reaction and programmed cell death by modulating the generation of reactive oxygen species (ROS) at the infection site and thereby restricting the penetration, invasion and further colonization of RKN (Nahar et al., 2011; Kyndt et al., 2012a,b). Similar results were reported on the MAPK-mediated defense-priming in soybean (*Glycine max*) in response to *Heterodera glycines* infection (Bekal et al., 2003; Zhang et al., 2017; McNeece et al., 2019) and in rice against *M. graminicola* (Kumari et al., 2016; Zhou et al., 2020). Asai et al. (2002) clearly demonstrated the role of MAPKs signaling cascades (*MEKK1, MKK4/MKK5*, and *MPK3/MPK6*) and WRKY transcription factors (*WRKY22/WRKY29*) in the activation of innate immune responses in *Arabidopsis thaliana*. It was further reported that *AtMPK3* and *AtMPK6* are involved in ETI in *Arabidopsis thaliana* (Tsuda et al., 2009; Meng and Zhang, 2013; Choi et al., 2017; Su et al., 2018; Zhang et al., 2018) which was further endorsed by several other researchers (Pitzschke et al., 2009; Rasmussen et al., 2012; Meng and Zhang, 2013). Similarly, qRT-PCR analysis clearly indicated that RKN infection suppresses the expression of CIPKs genes (−0.75 to 4.60 fold). However, inoculation of AM fungi up-regulated the CIPKs genes in roots of susceptible and resistant inbred lines (1.20 to 9.67 fold). CIPK9 repression augmented the total RKN population in roots of susceptible inbred lines. Previous reports clearly indicated that calcium sensor (Cbl10) together with its interacting partner *CIPK6* regulates plant immunity in tomato plants (De la Torre et al., 2013; Zhu et al., 2019; Huang et al., 2020). Similarly, *CIPK9* regulate the downstream signaling in rice to RKN invasion (Zhou et al., 2020). Further, RKN infection significantly altered the expression level of key genes involved in the BR biosynthesis and downstream signaling in rice root. Manifolds suppression was reported in the expression of the gene involved in the BR signaling in susceptible inbred line PB-1 as compared to the resistant line, Jasmine 85. However, AM inoculation up-regulated the BR biosynthesis genes in both susceptible and resistant inbred lines at 30 DAPI signifying the presence of diverse BR functions responding to RKN infection and disease development (Song et al., 2018). Our findings clearly signify a negative correlation between the activation of BR biosynthesis genes and RKN population on rice roots. These findings are also in agreement with the observation of earlier researchers (Kumari et al., 2016; Song et al., 2018; Zhou et al., 2020). In the present investigation, it was observed that plants inoculated with AM fungi showed significantly higher accumulation of antioxidants and defense-related mediator molecules/enzymes in them leading to increased cell wall lignification and reduction in the RKN invasion. qRT-PCR results also showed that RKN invasion negatively regulates the jasmonates biosynthesis genes, *OsAOS2, OsJMT1*, and *OsJAMYB*. However, these genes were highly up-regulated in the AM-inoculated plant which clearly indicated that AM inoculation has a positive impact on JA-mediated defense priming in rice (Seo et al., 2001; Kyndt et al., 2012a,b; Liu et al., 2013; Li et al., 2015; Zhou et al., 2020). Similar to BR biosynthesis and signaling, jasmonate biosynthesis and accumulation of jasmonic acids significantly affect the phenolics content in many crops (Wang and Zheng, 2005; Heredia and Cisneros-Zevallos, 2009). In the present study, the key genes involved in the ET biosynthesis (*OsACS1, OsACO7, OsEIN2*, and *OsERF1*) were suppressed upon RKN invasion in rice, while AM inoculation up-regulated these genes in resistant and susceptible inbred lines. ET biosynthesis and ET-mediated downstream signaling positively regulate the defense cascades in crop plants under biotic and abiotic stresses (Leon-Reyes et al., 2009; Molinari et al., 2014; Yuan et al., 2018; Zhou et al., 2020). Along with defense cascades, ET biosynthesis play a crucial role in IAA-mediated lateral root development, tissue differentiation, photosynthesis and overall plant growth and development. Earlier reports also emphasized that ET signaling is known to regulate plant response to RKN invasion (Yuan et al., 2018; Zhou et al., 2020).

Despite being stress-inducible, systemic mRNA levels of pathogenesis-related proteins, *OsPR-1, OsPR-2, OsPR-5*, and *OsPR-10* did not significantly change in the roots inoculated with AM fungi individually and pre-challenged with *M. graminicola* (Xiong and Yang, 2003; Leon-Reyes et al., 2009; Goto et al., 2013; Goverse and Smant, 2014). In contrast, significantly higher up-regulation was observed in the roots inoculated with consortia of AMF. The systemic mRNA levels of *OsPR-1, OsPR-2, OsPR-5*, and *OsPR-10* were consistently suppressed in the roots of PB-1 under RKN invasion at 30 DAPI (Figure 7). Among all defense mechanisms, PR proteins are the indispensable component of innate immune responses in plants under biotic or abiotic stress conditions (Sarma et al., 2015; Lv et al., 2016; Yuan et al., 2018; Zhou et al., 2020). The biosynthesis and accumulation of PR proteins protect the plants from further infection by not only accumulating locally in the infected and surrounding tissues but also in remote uninfected tissues. PR proteins are also involved in the synthesis of phytoalexins, regulation of oxidative burst, HR response or SA-mediated systemic acquired resistance against pathogenic infection including phytopathogenic nematodes (Harman et al., 2004; Sanz-Alferez et al., 2008; Pieterse et al., 2009; Vlot et al., 2009; Singh et al., 2020). Further structure, biochemistry, source, regulation of gene expression and nature of stress, define their role in defense mechanism of various PR proteins (Sanz-Alferez et al., 2008; Bari and Jones, 2009; Hamamouch et al., 2011; Kumari et al., 2016). Phenylpropanoid pathway is the first line of plant defense imparting host resistance by reprogramming the downstream signaling involving activation and accumulation of antioxidant enzymes (PAL, TAL, Pox, APx, etc.) and various defense-related biomolecules such as phytoalexins, callose, pectin, lignin derivatives and other metabolites toxic to the pathogens (Wuyts et al., 2006, 2007; Singh U. B. et al., 2019; Singh et al., 2020). Cell wall lignifications and deposition of pectin substances between the cells give mechanical strength to the plant tissues (Singh et al., 2020). In the present study, results clearly showed that RKN invasion repressed the expression of key genes of phenylpropanoid cascades and took benefit of that during the penetration and invasion of the root tissues. At the same time, plants bioprimed with AM fungi alone or in combination upregulated the expression profile of key genes of phenylpropanoid pathways and increased the activities of PAL, TAL, Pox, APx, etc. While in the resistant inbred line, the fold expression was significantly higher as compared to the susceptible cultivar. Similar effects were also observed in terms of the expression profile of key genes involved in lignin and callose biosynthesis (Wuyts et al., 2006, 2007; Hao et al., 2008; Portillo et al., 2013). Increased lignifications and callose deposition in response to RKN invasion provide additional adoptive mechanisms and strength to resistant inbred lines. Due to the antimicrobial and non-degradable nature of lignin, pectin and callose restrict the entry of pathogens including phytopathogenic nematodes and protect plants even under conducive conditions (Dixon and Paiva, 1995; Wuyts et al., 2006, 2007; Hao et al., 2008; Portillo et al., 2013; Singh U. B. et al., 2019; Singh et al., 2020).

**FIGURE 12:**
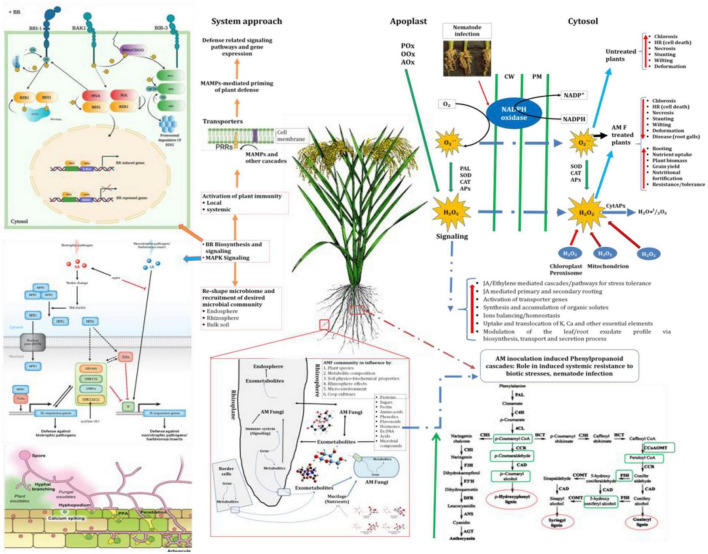
A schematic overview of AMF-mediated induction of defense cascades in rice under pathogenic stress of *M. graminicola*.

Apart from this, AMF have increased the accumulation of total chlorophyll, total soluble sugar and total protein in rice plants even under pathogenic stress. It is a well-established fact that AMF have the ability to mobilize diffusion-limited nutrients and water from distant places and supple to plants those helping plant growth and productivity (Opik et al., 2006; Parniske, 2008; Bagyaraj et al., 2022). Increased uptake and translocation of mineral nutrients contribute to improved total chlorophyll, total soluble sugar and total protein content in the plants which lead to better plant growth and vigor (Plett et al., 2014). It has also been reported that RKN invasion led to the overproduction of reactive oxygen species (ROS) at the cellular level (Singh U. B. et al., 2019; Singh et al., 2020). In the present investigation, manifold increases in the activities of antioxidant and defense-related enzymes were recorded in plants primed with consortia of three AMF *F. mosseae, R. fasciculatus* and *R. intraradices* compared to plants primed individually with each of them and conferring resistance to RKN invasion. Among them, ascorbate peroxidase, peroxidase, catalase, and superoxide dismutase are the most important. These antioxidant enzymes are known to reduce ROS more efficiently. These results are in agreement with the findings of other earlier researchers (Lorito et al., 2010; Singh et al., 2011). Unlike AM fungi, roots primed with plant growth-promoting microorganisms modulated different cascades related to defense related metabolome in plant systems over a time period and activated plant growth and defense biome directly and/or indirectly (Ahn et al., 2007; Bakker et al., 2013; Facelli et al., 2014; Alori et al., 2017; Singh A. et al., 2019; Singh U. B. et al., 2019). As revealed by infection bioassay in the present study (Table 1), a significantly higher number of galls/root system, egg mass/root system and J_2_s/root system was reported in the roots of susceptible inbred line, PB-1 compared to the resistant inbred line, Jasmine 85 during early stage of infection (30 DAPI). A similar trend was also recorded at 45 DAPI. It is well established that the resistant inbred line, Jasmine 85 contains several resistant genes/QTLs as compared to susceptible ones which protect plants from severe invasion and development of root gall (Ryan and Graham, 2002; Selosse and Rousset, 2011). Further, inoculation of AM fungi individually or in combination primed the plant and activated several genes involved in the plant defense which gives addition support to the plant defense even under RKN invasion. Increased lignifications and callose deposition restrict the entry of J_2_s and the invasion caused by them. AM inoculation not only reduced the RKN invasion but also increased plant growth attributes under the pathogenic stress of *M. graminicola.* In short, the present study describes well-coordinated mode of action of AM fungi during RKN invasion, expression of defense-related genes regulating several pathways, accumulation and activities of mediator molecules/enzymes, callose deposition and cell wall lignification ultimately leading to the reduction in RKN invasion, disease development and improved plant growth under biotic stress condition (Figure 12).

## Conclusion

Root priming with AMF, *F. mosseae, R. fasciculatus* and *R. intraradices* individually or in combination significantly increased the transcript accumulation of key genes involved in the signaling and plant defense in the susceptible and resistant inbred lines, PB-1 and Jasmine 85 pre-challenged with RKN, *M. graminicola*. The present study, has summarized the detailed investigation of AM fungi-induced activation and accumulation of defense-related biomolecules in rice plants even under pathogenic stress conditions. These AMF could be very promising microbial inoculants for the activation of defense pathways in a cooperative manner under pathogenic stress. Inoculation of AMF also increased lignin content in roots which further reduces the pathogen infection, colonization and invasion. Results clearly indicated that inoculation of AM fungi alone or in combination significantly reduced the number of gall/root system, egg mass/root system and J_2_s/root system at 30 and 45 DAPI. Inoculation of AM fungi to rice led to modifications in the root architecture that supports efficient uptake of nutrients and water and promotes plant growth even under pathogenic stressed conditions. With the help of these findings, we concluded that AMF inoculation could be a potential alternative to toxic chemical nematicides and could be applied at a larger scale to manage root-knot disease in rice at experimental plots and commercial production after extensive field evaluation at farmers’ fields.

## Data availability statement

The original contributions presented in this study are included in the article/Supplementary material, further inquiries can be directed to the corresponding authors.

## Author contributions

DM, PS, US, JR, and HS conceived and designed the experiments. DB supplied the AM fungi. PS, RV, and AK given the resistant and susceptible inbred lines/cultivars of rice. DM, PS, and US performed the experiments. PS, PKB, US, and RAF analyzed the data. DM, SP, PK, PS, and US wrote the manuscript. AK, DB, JR, MA, and SD edited and given final touch to the manuscript. All authors reviewed the manuscript and given approval to the final version.
