# Supplementary material for: Arbuscular mycorrhizal fungi-mediated activation of plant defense responses in direct seeded rice (Oryza sativa L.) against root-knot nematode Meloidogyne graminicola
Source: Front Microbiol. 2023 May 2;14:1104490. doi: 10.3389/fmicb.2023.1104490 (PMC10185796; doi:10.3389/fmicb.2023.1104490)
Supplement: Supplementary file 1 [file Data_Sheet_1.doc]

**Arbuscular Mycorrhizal Fungi-mediated activation of plant defense responses in direct seeded rice (*Oryza sativa* L.) against root-knot nematode *Meloidogyne graminicola***

Deepti Malviya^1,†^, Prakash Singh^2,†,*^, Udai B. Singh^1,†^, Surinder Paul^1^, Pradeep Kumar Bisen^3^, Jai P. Rai^4^, Ram Lakhan Verma^5^, R. Abdul Fiyaz^6^, Arun Kumar^7^, Poonam Kumari^8^, Sail Bala Dei^7^, Md. Reyaz Ahmed^7^, D.J. Bagyaraj^9^ and Harsh V. Singh^1*^

**Supplementary Figure 1.** Effect of *F. mosseae, R. fasciculatus* and *R. intraradices* inoculation on expression profile of key genes involved in BR biosynthesis in the (A) susceptible inbred line, PB-1 and (B) resistant inbred line, Jasmine 85 of rice pre-challenged with *M. graminicola* at 30 days of sowing under greenhouse conditions. Treatments were: T_1_- *M. graminicola,* T_2_- *M. graminicola* + *F. mosseae,* T_3_- *M. graminicola* + *R. fasciculatus,* T_4_- *M. graminicola* + *R. intraradices,* T_5_- *M. graminicola* + *F. mosseae* + *R. fasciculatus* + *R. intraradices,* and T_6_- Control (untreated). Column data are mean (n = 5) and vertical bar represents standard deviation.

| (A) 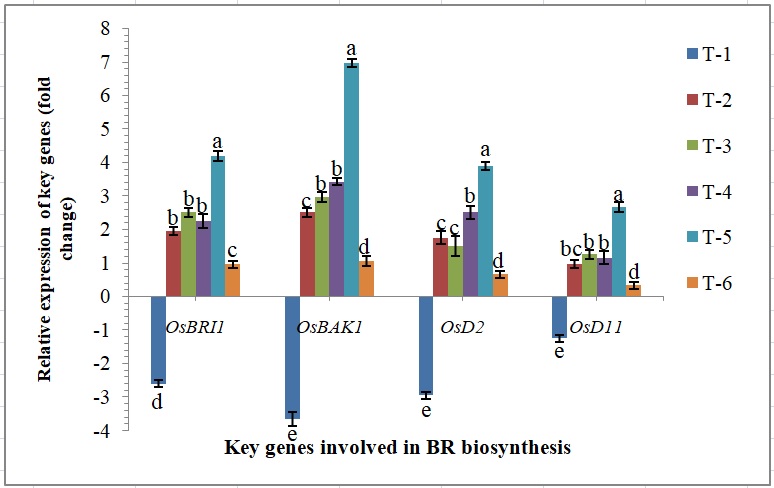 |
| --- |
| (B) 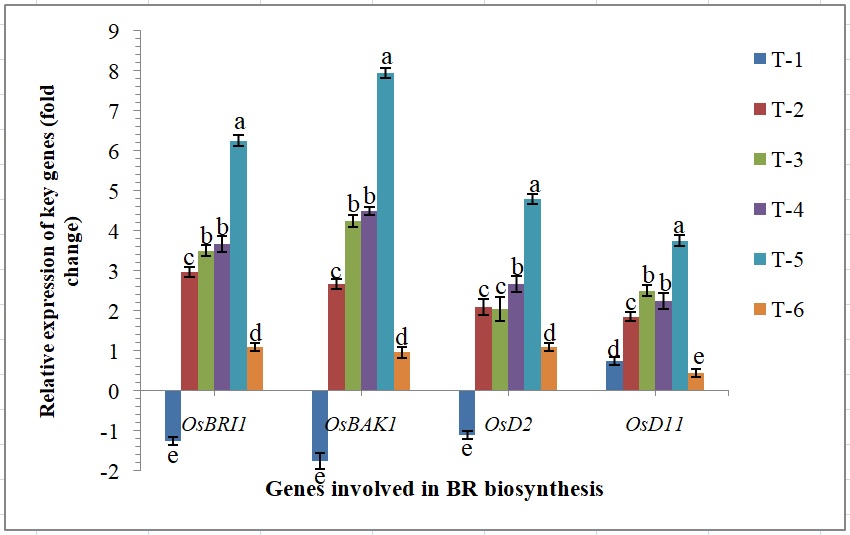 |

**Supplementary Figure 2. Effect of *F. mosseae, R. fasciculatus* and *R. intraradices* inoculation on expression profile of key genes involved in jasmonate biosynthesis in the (A) susceptible inbred line, PB-1 and (B) resistant inbred line, Jasmine 85 of rice pre-challenged with *M. graminicola* at 30 days of sowing under greenhouse conditions. Treatments were: T_1_- *M. graminicola,* T_2_- *M. graminicola* + *F. mosseae,* T_3_- *M. graminicola* + *R. fasciculatus,* T_4_- *M. graminicola* + *R. intraradices,* T_5_- *M. graminicola* + *F. mosseae* + *R. fasciculatus* + *R. intraradices,* and T_6_- Control (untreated). Column data are mean (n = 5) and vertical bar represents standard deviation.**

| (A) 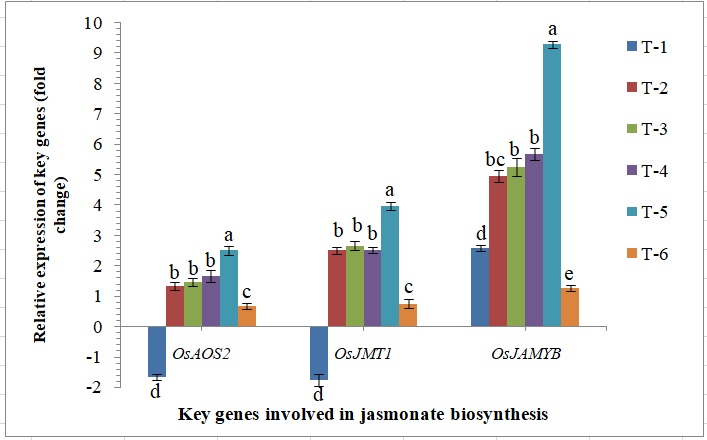 |
| --- |
| (B) 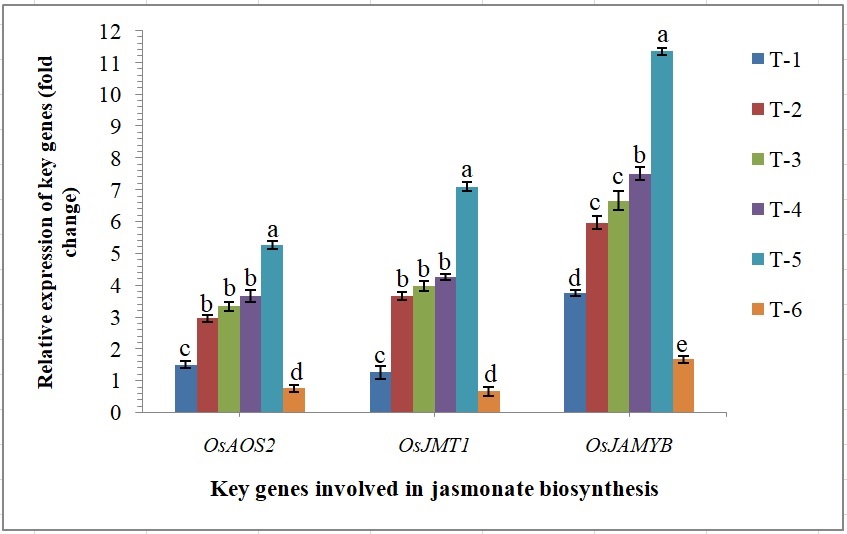 |

**Supplementary Figure 3.** Effect of *F. mosseae, R. fasciculatus* and *R. intraradices* inoculation on expression profile of key genes involved in ethylene biosynthesis in the (A) susceptible inbred line, PB-1 and (B) resistant inbred line, Jasmine 85 of rice pre-challenged with *M. graminicola* at 30 days of sowing under greenhouse conditions. Treatments were: T_1_- *M. graminicola,* T_2_- *M. graminicola* + *F. mosseae,* T_3_- *M. graminicola* + *R. fasciculatus,* T_4_- *M. graminicola* + *R. intraradices,* T_5_- *M. graminicola* + *F. mosseae* + *R. fasciculatus* + *R. intraradices,* and T_6_- Control (untreated). Column data are mean (n = 5) and vertical bar represents standard deviation.

| (A) 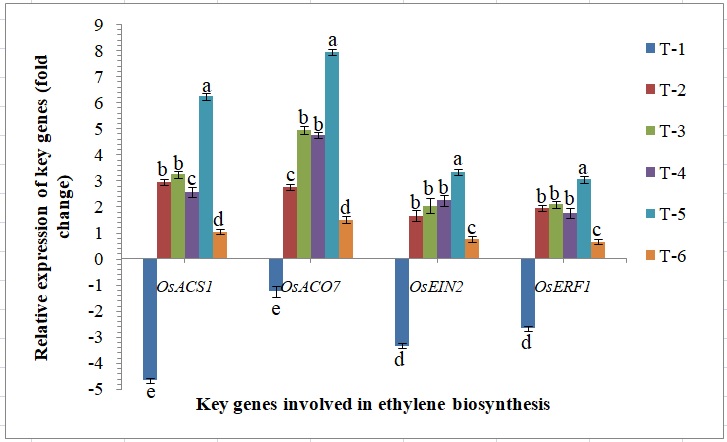 |
| --- |
| (B) 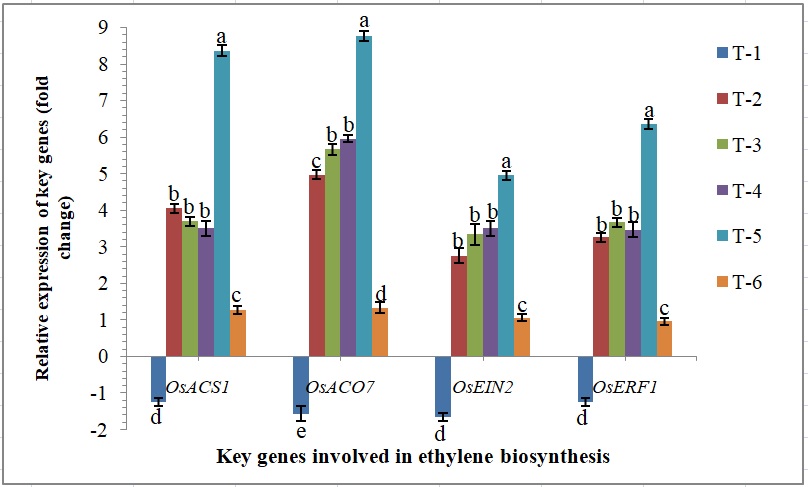 |
