# Supplementary material for: Arbuscular mycorrhizal fungi-mediated activation of plant defense responses in direct seeded rice (Oryza sativa L.) against root-knot nematode Meloidogyne graminicola
Source: Front Microbiol. 2023 May 2;14:1104490. doi: 10.3389/fmicb.2023.1104490 (PMC10185796; doi:10.3389/fmicb.2023.1104490)
Supplement: Supplementary file 2 [file Table_1.docx]

**Arbuscular Mycorrhizal Fungi-mediated activation of plant defense responses in direct seeded rice (*Oryza sativa* L.) against root-knot nematode *Meloidogyne graminicola***

Deepti Malviya^1,†^, Prakash Singh^2,†,*^, Udai B. Singh^1,†^, Surinder Paul^1^, Pradeep Kumar Bisen^3^, Jai P. Rai^4^, Ram Lakhan Verma^5^, R. Abdul Fiyaz^6^, Arun Kumar^7^, Poonam Kumari^8^, Sail Bala Dei^7^, Md. Reyaz Ahmed^7^, D.J. Bagyaraj^9^ and Harsh V. Singh^1*^

Supplementary Table 1. Primers of key genes of MAPKs pathways used in expression analyses.

| **S. No.** | **Gene** | **Primer sequences** | | **Primer efficiency (%)** | **Primer source** |
| --- | --- | --- | --- | --- | --- |
| A. Key genes involved in the signaling process | | |  |  |  |
|  | *OsCERK1* | F- TCCGGGAGATAGTGACTTTGGT | R- GGCTGTCATTACACACTTCAGATGA | 98 | Huang et al., 2020 |
|  | *OSCEBiP* | F- CACCATGGCGTCGCTCACCGCCGC | R- TCAAAGGAAACAGATAATGA | 96 | This study |
|  | *OsWRKY25* | F- CGTCGTCGTCTCCTGTCTC | R- GATTCGTGAAGTCCAGCGT | 99 | Choi et al., 2015 |
|  | *OsWRKY29* | F- ACCTACGAGGGCCAACACT | R- TGCAGGTTGCTGTACAGTTG | 96 | Choi et al., 2015 |
|  | *OsWRKY70* | F- ACTCTTACACGAGCCAGCAG | R- ACATGATGCCGTCGTCTCTC | 97 | Choi et al., 2015 |
|  | *OsMYB15* | F- GGAGGGTGATCACAAGTCGA | R- ACAACATGCACCATCTACGC | 98 | This study |
|  | *OsMAPKKK1* | F- GAGTGAAGTGCGGATTATG | R- TTGGAGGACGAGTAACAG | 95 | Yang et al., 2015 |
|  | *OsMAPKKK5* | F- CCTCAGTGGACATCACTTATCC | R- CTCGAAGTCTCTCCAGAAGTTG | 98 | Yang et al., 2015 |
|  | *OsMAPK5* | F- TCCGTGATGAACTTTGAG | R- GTCCATGTCGTTGTTGAA | 98 | Yang et al., 2015 |
|  | *OsMAPK6* | F- TTGCTACGAGGGCTAAAATATGTG | R- GGAACAAATTGCTTGGCTTCA | 96 | Yang et al., 2015 |
|  | *OsMAPK15* | F- TTGAAGAACATTATGCTAAGG | R- CATCCGAATATAACACAGAAG | 97 | Yang et al., 2015 |
|  | *OsMAPK17* | F- AACCTGACTTCTTCACTGA | R- GCCACAACACCATAACTC | 96 | Yang et al., 2015 |
| B. Key calcineurin B-like protein-interacting protein kinases genes involved in signaling | | | | | |
|  | *OsCIPK5* | F- TTCTGCAACAAGGGCCTAAG | R- TCAGGCTCAGCACTTTGATG | 99 | Zhou et al., 2020 |
|  | *OsCIPK8* | F- GTCAAGCATGGAGGTTGTTG | R- GTTATAATCGGAGGTGTCTC | 99 | Zhou et al., 2020 |
|  | *OsCIPK9* | F- TGAGCTTCGTAAAACTGGAG | R- GCATTGGATAGTTGGAATAC | 96 | Zhou et al., 2020 |
|  | *OsCIPK11* | F- AAGGATATTGTTTGGGTGTG | R- TGTTTGTTCTGCGGGGATAG | 99 | Zhou et al., 2020 |
|  | *OsCIPK14* | F- GAAGGAATGGTGTTCTTCAG | R- ATCTACTCTTGCGACTGCTG | 98 | Zhou et al., 2020 |
|  | *OsCIPK23* | F- TGGGCTTTAATGTACAGAAG | R- TCACATCTTTCAGGCCATTG | 96 | Zhou et al., 2020 |
|  | *OsCIPK24* | F- TGTTGCTGAGACTATGGGTC | R- GAACCTGTGGTATTCCAGTG | 97 | Zhou et al., 2020 |
|  | *OsCIPK31* | F- AGTAGCTCCATCCTTACATG | R- TGGCAAAAACCACGTTCACG | 97 | Zhou et al., 2020 |
| C. Key genes involved in the BR signaling and regulation | | | | | |
|  | *OsBRI1* | F- CAGCTACTTGGCTATCTTGAAGCTCAGC | R- CCATTCTTGTTGAAGGTGTACTCCGTGC | 95 | Zhou et al., 2020 |
|  | *OsBAK1* | F- TTTCCTTGTGCATGCTAG | R- GCATCTCCATAATTGATG | 94 | Zhou et al., 2020 |
|  | *OsD11* | F- AGTGAAGAGGGAGCATGAAGGCAT | R- ATCTGCAGGGCTGAAATTGTTGGG | 98 | Zhou et al., 2020 |
|  | *OsD2* | F- ATGTGATAACAGAGACGCTGCGGT | R- TGGTGACCAAGTGGTGAAGGAAGA | 98 | Zhou et al., 2020 |
| D. Key genes involved in the jasmonate biosynthesis | | | | | |
|  | *OsAOS2* | F- GCGAGAGACGGAGAACCC | R-CGACGAGCAACAGCCTTC | 98 | Kumari et al., 2016 |
|  | *OsJAMYB* | F- GAGGACCAGAGTGCAAAAGC | R- CATGGCATCCTTGAACCTCT | 96 | Kumari et al., 2016 |
|  | *OsJMT1* | F- CACGGTCAGTCCAAAGATGA | R- CTCAACCGTTTTGGCAAACT | 99 | Kumari et al., 2016 |
| E. Key genes involved in the ethylene biosynthesis | | | | | |
|  | *OsACS1* | F- GATGGTCTCGGATGATCACA | R- GTCGGGGGAAAACTGAAAAT | 99 | Kumari et al., 2016 |
|  | *OsACO7* | F- GGACTACTACCAGGGCACCA | R- GATTAGCGCACGCGATTTTA | 98 | Kumari et al., 2016 |
|  | *OsEIN2* | F- TAGGGGGACTTTGACCATTG | R- TGGAAGGGACCAGAAGTGTT | 99 | Kumari et al., 2016 |
|  | *OsERF1* | F- AAGGGTCATAATTCGCGTCA | R- TCCACACCACAAGACATCGT | 99 | Kumari et al., 2016 |
| F. Key genes involved in the general defense | | | | | |
|  | *OsPR1* | F- AACTTCGTCGGCCAATCTC | R- CATGCATAAACACGTAGCATAGC | 96 | Kumari et al., 2016 |
|  | *OsPR2* | F- TGCTATGTTCGACGAGAACG | R- GTTGAACAGCCCAAAGTGCT | 95 | This Study |
|  | *OsPR5* | F- CAGCCAGGACTTCTACGA | R- TGTGTCTTGGTGTTGTCTTC | 96 | Lv et al., 2016 |
|  | *OsPR10* | F- ACGCCTAAGATGAAGAGGAATAC | R- CTCAAACGCCACGAGAATTTG | 99 | Kumari et al., 2016 |
| G. Key genes involved in the phenylpropanoid pathway | | | | | |
|  | *OsPAL* | F- TGTGCGTGCTTCTGCTGCTG | R- AGGGTGTTGATGCGCACGAG | 98 | This study |
|  | *OsTAL* | F- ACATCGGCAAGCTCATGTTC | R- CATGGTTGGTGATGGGGTTG | 98 | This study |
|  | *Os4-CL* | F- AGGATGATCTTGCCGGTGAA | R- TGGCTCTCAAGTCCTTCCTG | 99 | This study |
|  | *OsCCR* | F- GCTCATTTGCATGCTCTGGA | R- TGGCAGAAGTTCAGGGTCAT | 97 | This study |
|  | *OsCAD* | F- CGACTCGCTGGACTACATCA | R- AGAAGTTGAGCACCTCCTCC | 97 | This study |
|  | *OsPOx* | F- TGTCCGACAGCTACTACGAC | R- CAGTGTCGTCAAGAAGCACC | 99 | This study |
|  | *OsF5H* | F- TCCAGGAGTTCTCCAAGCTG | R- CCATGTGCTCGTCGATGATC | 99 | This study |
|  | *OsCCoAOMT* | F- TCCTCAAGAGCGACGATCTC | R- ACGTCGTCATCAGGTTCCAT | 99 | This study |
|  | *OsCALDH* | F- CGAGATGGCCAAGAAATCGG | R- AGATGGTGGGCTCGATGTAG | 98 | This study |
| H. Key genes involved in the lignin and callose biosynthesis | | | | | |
|  | *OsC4H* | F- CAGACTGGTGAGATCCGGTG | R- TTCCCCATTCGATCGACCAC | 96 | Kumari et al., 2016 |
|  | *OsCAD6* | F- TCGGTAAGAGGACGGTGAGT | R- TGTCGATGTCCCAGGTGATG | 95 | Kumari et al., 2016 |
|  | *OsGSL1* | F- TGAGGACCTGCCACGATT | R- CACGCTGATTGCGAACAT | 95 | Kumari et al., 2016 |
|  | *OsGSL3* | F- TGGCAAGCGACCACATAG | R- AGACCTTAGCACGGACTG | 96 | Kumari et al., 2016 |
|  | *OsGSL5* | F- GTGGTGTCCCTGCTATGA | R- GTTGTTTGCTATTCTCCC | 94 | Kumari et al., 2016 |
|  | *OsGNS5* | F- TTGCGGCCATTCCTACAGT | R- TGGTGAGGGCGATGCTTG | 96 | Kumari et al., 2016 |
| I. Reference genes/Housekeeping genes | | | | | |
|  | *OsACTIN* | F- CAATCGTGAGAAGATGACCC | R- GTCCATCAGGAAGCTCGTAGC | 98 | This study |
|  | *OsGAPDH* | F- GATAACTTTGTCAAGCTTGTCGC | R- CAGCATAGACAAAGCATACCGG | 97 | This study |
